# The distinct landscape of tumor immune microenvironment in homologous recombination deficient cancers

**DOI:** 10.1186/s40364-025-00814-x

**Published:** 2025-08-20

**Authors:** Qiuyang Xu, Yuanjia Wen, Taiyuan Huang, Huayi Li, Xingzhe Liu, Shen-nan Shi, Wenjian Gong, Gordon B. Mills, Ding Ma, Qinglei Gao, Yong Fang

**Affiliations:** 1 National Clinical Research Center for Obstetrics and Gynecology, Cancer Biology Research Center (Key Laboratory of the Ministry of Education), Tongji Hospital, Tongji Medical College, Huazhong University of Science and Technology, Wuhan, China; 2 Department of Gynecological Oncology, Tongji Hospital, Tongji Medical College, Huazhong University of Science and Technology, Wuhan, China; 3 Knight Cancer Institute, Oregon Health & Science University, Portland, OR USA; 4 Department of Cardiology, The First Affiliated Hospital of Chongqing Medical University, Chongqing, China

**Keywords:** Tumor immune microenvironment, Homologous-recombination deficient, PAPR inhibitor, Immune checkpoint inhibitors

## Abstract

Homologous recombination deficiency (HRD) is a common characteristic of human cancers, which occurs most frequently in ovarian and breast cancers. The unique genetic vulnerabilities, coupled with the synthetic lethality effect of Poly-(ADP-ribose) polymerase (PARP) inhibitors (PARPi), provide a promising opportunity for targeting HRD cancers. However, only a few HRD patients benefit from PARPi monotherapy, and it is therefore imperative to explore effective combination therapies for HRD tumors. Growing evidence has underscored the distinct tumor microenvironment (TME) landscape of HRD cancers. Immune activation and immune suppression co-exist in a dynamic balance during the development of HRD cancers. At the late stage, however, negative immune regulation predominates, resulting in the formation of an immunosuppressive microenvironment. This intricate network reprograms cancer biology in multiple aspects and serves as a potential target for cancer treatment. In this review, we briefly outline the current HRD tests and genetic characteristics of HRD cancers, focusing on breast, ovarian, pancreatic, and prostate cancers. We then summarize the interactions and crosstalk between immune cells and cancer cells, as well as various signaling pathways within the TME of HRD cancers. Additionally, we highlight recent advances in combining PARP inhibitors with immunotherapies in preclinical models and clinical trials of HRD cancers. This review provides valuable insights and perspectives into the distinct landscape of TME in HRD cancers, and offers a rationale for expanding the application of this combined therapeutic approach to a broader range of HRD cancers.

## Background

DNA damage is repaired through an intricate network of interrelated pathways, one of which is the homologous recombination repair (HRR) pathway [1]. The HRR pathway functions as a high-fidelity system for repairing double-strand breaks (DSBs), ensuring genomic integrity [2]. HRD refers to cellular dysfunction in the HRR process. In HRD cells, DSB repair relies on alternative, less accurate repair mechanisms such as non-homologous end joining (NHEJ) [3], microhomology-mediated end joining (MMEJ) [4], or single-strand annealing (SSA) [5]. These error-prone pathways can compromise genomic and chromosomal stability [6, 7]. HRD arises from various factors, including germline or somatic mutations and epigenetic modifications in HRR-related genes [8]. Among these, BRCA1 and BRCA2 stand out as the most critical components of the HRR pathway [9]. Carriers of germline BRCA1/2 mutations harbor a significantly elevated risk of developing breast, ovarian, pancreatic, and prostate cancers [10]. Mutations in other key HRR genes, such as ATM, RAD51, and PALB2, could induce a BRCAness phenotype characterized by HRD [11–14]. HRD is a prevalent feature in various malignancies, particularly in breast and ovarian cancers [15–17]. Recent studies have also demonstrated that a substantial proportion of pancreatic and prostate cancer patients harbor mutations in key HRR genes, notably BRCA1/2, ATM, PALB2, and CDK12 [18–20].

In 2005, two landmark studies demonstrated that tumor cells deficient in BRCA1 or BRCA2 exhibit selective sensitivity to small-molecule inhibitors of the PARP family. These findings first introduced the concept of synthetic lethality with PARP inhibitors [21, 22]. HRD renders tumor cells more sensitive to platinum-based agents that induce DNA damage and enhances the anti-tumor efficacy of PARPi [23, 24]. Currently, HRD is being developed as a critical biomarker for precision oncology therapies. Extensive preclinical and clinical studies have confirmed the effectiveness of PARPi in patients with various cancers harboring germline or somatic BRCA mutations, leading to FDA approval of PARPi for the treatment of ovarian, breast, prostate, and pancreatic cancers [25–28]. Furthermore, alterations in HRR pathway genes have been observed in other malignancies, including melanoma [29, 30], endometrial cancer [31, 32], cholangiocarcinoma [33, 34], gastric cancer [35], and non-small cell lung cancer (NSCLC) [36, 37], suggesting a broader potential application of PARPi across multiple cancer types.

Multiple studies have demonstrated that HRD cancers exhibit a distinct TME [38, 39]. Due to their reliance on low-fidelity DNA repair mechanisms, HRD cancers accumulate a higher tumor mutation burden (TMB), leading to an increased level of neoantigens [40]. This accumulation serves as a potent signal for activating anti-tumor immunity. In the TME of HRD cancers, antigen-presenting cells (APCs) become active, initiating T cell activation and subsequently enabling immune surveillance [41]. However, recent evidence indicates that while HRD cancers exhibit an immune activation status, they also develop an immunosuppressive phenotype characterized by the upregulation of co-inhibitory molecules such as PD-1, TIM-3, LAG-3, and TIGIT, which suggests the enrichment of functionally exhausted immune cells [42, 43]. Moreover, an increased amount of immunosuppressive cells within the TME, including regulatory T cells (Tregs) and M2-type macrophages, is also observed [44, 45]. An improved understanding of this unique TME landscape with concurrent immune activation and suppression can provide deeper insights into HRD cancers and inform the clinical application of PARPi in combination with immunotherapies.

In this review, we focus on HRD phenotypes in ovarian, breast, prostate, and pancreatic cancers, summarize the genetic characteristics of HRD cancers, and dissect the interactions and crosstalk among various cells, cytokines, and signaling pathways within the TME. We also highlight recent advances in preclinical models and clinical trials assessing the combination of PARPi and immunotherapies for HRD cancers.

## HRD tests and genetic characteristics of HRD cancers

### HRD tests

The evaluation of HRD in clinical settings serves as a critical tool for guiding the administration of PARPi and other DNA damage agents. Consequently, a thorough understanding of the details and limitations of these detection methods is essential to ensure their optimal clinical application. Currently, HRD testing is approved by the FDA only for ovarian cancer, while determining appropriate treatments for prostate, pancreatic, and breast cancers also seems to be important [46]. Clinically, HRD testing methods are primarily categorized into three types: germline or somatic mutation test in HR genes, genomic signature and scar measurement, and functional assays of HRD.

#### Germline or somatic mutations of HR genes

**Fig. 1:**
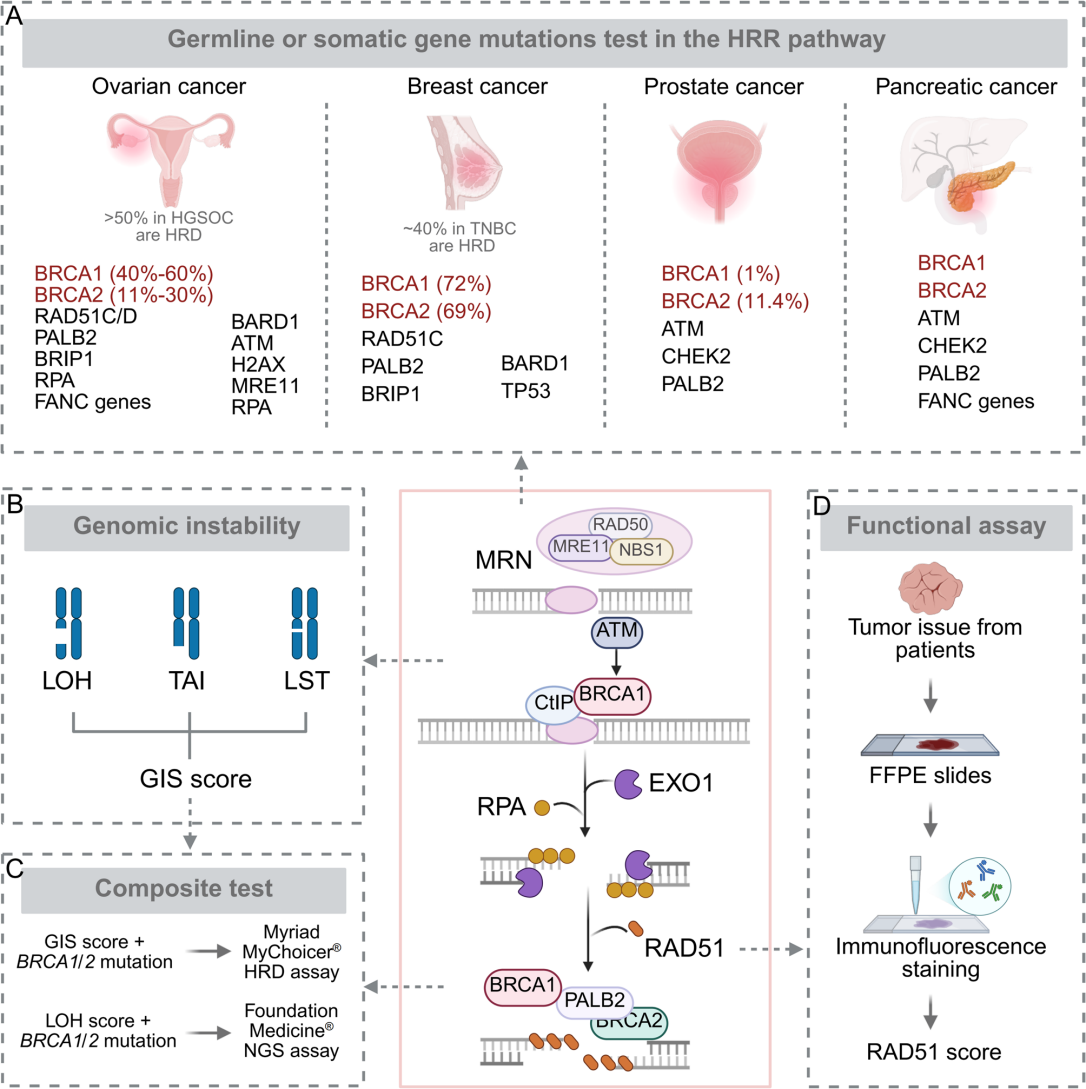
HRD tests and genetic characteristics of HRD cancer. HRD testing methods are primarily categorized into three types: germline or somatic mutation test in HR genes (**A**), genomic signature and scar measurement (**B** and **C**), and functional assays of HRD (**D**). Different genetic characteristics exist among different HRD cancers. HRR, homologous recombination repair; HRD, homologous recombination deficiency; FFPE, Formalin-Fixed and Paraffin-Embedded; LOH, loss of heterozygosity; TAI, telomeric allelic imbalance; LST, large scale transitions; GIS, genomic instability scores; HGSOC, high grade serous ovarian cancer; TNBC, triple-negative breast cancer

The BRCA1/2 proteins play a critical role in HRR of DSBs [47]. The development of PARPi as the treatment for high-grade serous ovarian cancer (HGSOC) patients stems from observations that BRCA mutations significantly enhance the in vitro sensitivity of cancer cells to PARP inhibition [21, 22]. A recent study has revealed that germline BRCA1 and BRCA2 (gBRCA) mutations occur in 13%-15% of HGSOC cases [48]. Consequently, the most direct and classical detection method involves assessing germline mutations in the BRCA1/2 genes to identify patients who would benefit from PARPi maintenance therapy (Fig. 1A).

However, approximately 5% of patients with gBRCA mutations exhibit negative outcomes in BRCA tests with tumor samples. Supplementary somatic BRCA (sBRCA) testing can identify an additional 6–7% of these patients who acquire BRCA mutations during cancer development or progression [49]. Although the evidence on sBRCA as a biomarker to determine the benefit of PARPi is more limited compared with that for gBRCA, clinical trials have demonstrated that patients with sBRCA mutations exhibit similar progression-free survival (PFS) outcomes to those with gBRCA mutations [50, 51].

In addition to BRCA1/2, defects in other genes within the HR pathway may also result in the BRCAness phenotype. Preclinical studies have demonstrated that deficiencies in genes such as ATM, CDK12, and RAD51 confer sensitivity to DNA repair inhibition [52, 53]. In a previous study, two patients harboring mutations in RAD51C and RAD51D achieved clinical benefits from rucaparib treatment, a PARPi [54]. However, given the relative rarity of these gene defects compared with BRCA1/2 mutations, greater caution is required when using them as biomarkers to predict the efficacy of PARPi.

#### Genomic signature and scars

The tumor cells of HRD patients are unable to utilize the HR pathway for DSB repair, and consequently rely on the NHEJ pathway. Since NHEJ does not depend on sister chromatids for repair, it is an error-prone process that leads to characteristic “genomic scars”, which serve as a hallmark of HRD cancers [55]. The quantification of these “genomic scars” has become an attractive topic in recent cancer genomics research. Currently, predicting HRD status primarily involves measuring three types of genomic alterations: large-scale transitions (LST), loss of heterozygosity (LOH), and telomeric allelic imbalance (TAI) (Fig. 1B) [56, 57]. Evaluating these three genomic aberrations can improve the identification of HRD-positive samples [58, 59]. To date, the most commonly reported methods for detecting genomic scars are two commercially available tests. These tests integrate the tumor BRCA mutation detection with the genomic instability score (GIS), which is derived from the total score of TAI, LST, and LOH (myChoice HRD test, Myriad Genetics), or the subchromosomal LOH fraction (FoundationFocus CDxBRCA, Foundation Medicine) (Fig. 1C) [60, 61].

#### Functional assays of HRD

The most widely used method for assessing HR functional status is detecting nuclear RAD51 (Fig. 1D) [62, 63]. RAD51 facilitates high-fidelity double-strand DNA repair through its interaction with the BRCA1/PALB2/BRCA2 complex [64]. In preclinical models and patient samples of ovarian and breast cancer, including ascite metastases and in situ carcinomas, reduced DNA damage-induced nuclear RAD51 is positively associated with BRCA1 or BRCA2 gene defects and patient response to PARPi [65, 66]. In breast cancer, lower levels of RAD51 positively correlate with responses to neoadjuvant chemotherapy or PARPi treatment [67, 68]. However, further validation in larger clinical cohorts is necessary to establish the clinical utility of nuclear RAD51 detection.

### Genetic characteristics of HRD cancers

HRD gives rise to impaired DSB repair, which constitutes a common driver for cancer development. The analysis of large metastatic and primary cancer cohorts reveals that HRD is most prevalent in ovarian and breast cancer, followed by pancreatic and prostate cancer [39]. Notably, different genetic characteristics also exist among these four tumors (Fig. 1A).

#### Ovarian cancer

Ovarian cancer ranks as the third most prevalent cancer in the female reproductive system and holds the highest mortality rate among gynecological malignancies [69]. Among all types of ovarian cancer, 75% are HGSOC, and over 50% of HGSOC present HRD status [70, 71]. Among all cancers, both the germline and somatic mutation frequencies of BRCA1 or BRCA2 in HGSOC are the highest [72]. Additionally, defects in other HR-related genes, including RAD51C/D, PALB2, ATM, H2AX, MRE11, RPA, BRIP1, BARD1, and FANC genes, that can confer HRD or BRCAness phenotypes exist in HGSOC patients [73]. Therefore, PARPi maintenance therapy has been incorporated into clinical treatment guidelines for HRD ovarian cancer patients.

#### Breast cancer

Breast cancer is the most prevalent malignant tumor among women and the fourth leading cause of cancer-related deaths [74]. Deficiencies in the DNA damage repair pathway related genes constitute an important factor in the development of breast cancer [75], with approximately 10% of breast cancer cases being caused by mutations in HR genes, such as BRCA1, BRCA2, PALB2, BRIP1, BARD1, and RAD51C [76, 77]. The proportion of HRD is higher in triple-negative breast cancer (TNBC), with nearly 40% of TNBC samples being marked as HRD. TP53 mutations are significantly enriched in HRD samples, which is consistent with their usual co-occurrence with the most common BRCA1/2 defects. Additionally, chromosome arm 16p is significantly enriched in HRD samples, as it carries the PALB2 gene. PALB2 is a highly important protein in the HR pathway, and is closely associated with the occurrence of breast cancer and the sensitivity of PARPi treatment [78].

#### Prostate cancer

Patients with the impaired HRR pathway constitute a considerable portion of the prostate cancer population [79]. BRCA2, ATM, and CHEK2 are the three most common DNA repair mutation genes, accounting for approximately three-quarters of all DNA repair gene mutations in the disease, both at the germline and somatic levels [80, 81]. Besides these three genes, PALB2 germline mutations are also associated with an increased risk of prostate cancer [82].

#### Pancreatic cancer

Owing to the restricted treatment options, pancreatic ductal adenocarcinoma (PDAC) is among the most lethal solid malignant tumors [83]. A growing body of evidence indicates that HRD serves as a biomarker to select the intended population for PARPi maintenance therapy in late-stage platinum-sensitive PDAC patients [84]. The HRD phenotype in PDAC is predominantly orchestrated by mutations in BRCA2, BRCA1, and ATM, followed by FANC genes, CHEK2, and PALB2. The mutation rate of RAD51, ATR, BRIP1, BARD1, CDK12, and CHEK1 is significantly lower [85]. Furthermore, pancreatic acinar cell carcinoma (PACC) has also been reported to be correlated with HRD, with a significantly higher prevalence of HR/DDR germline genetic alterations in BRCA1, BRCA2, PALB2, ATM, and CHEK2 in PACC compared with other tumors, among which BRCA2 mutation occurs most frequently [44].

## The distinctive phenotypes and Spatial connection of cells in the TME of HRD cancers

**Fig. 2:**
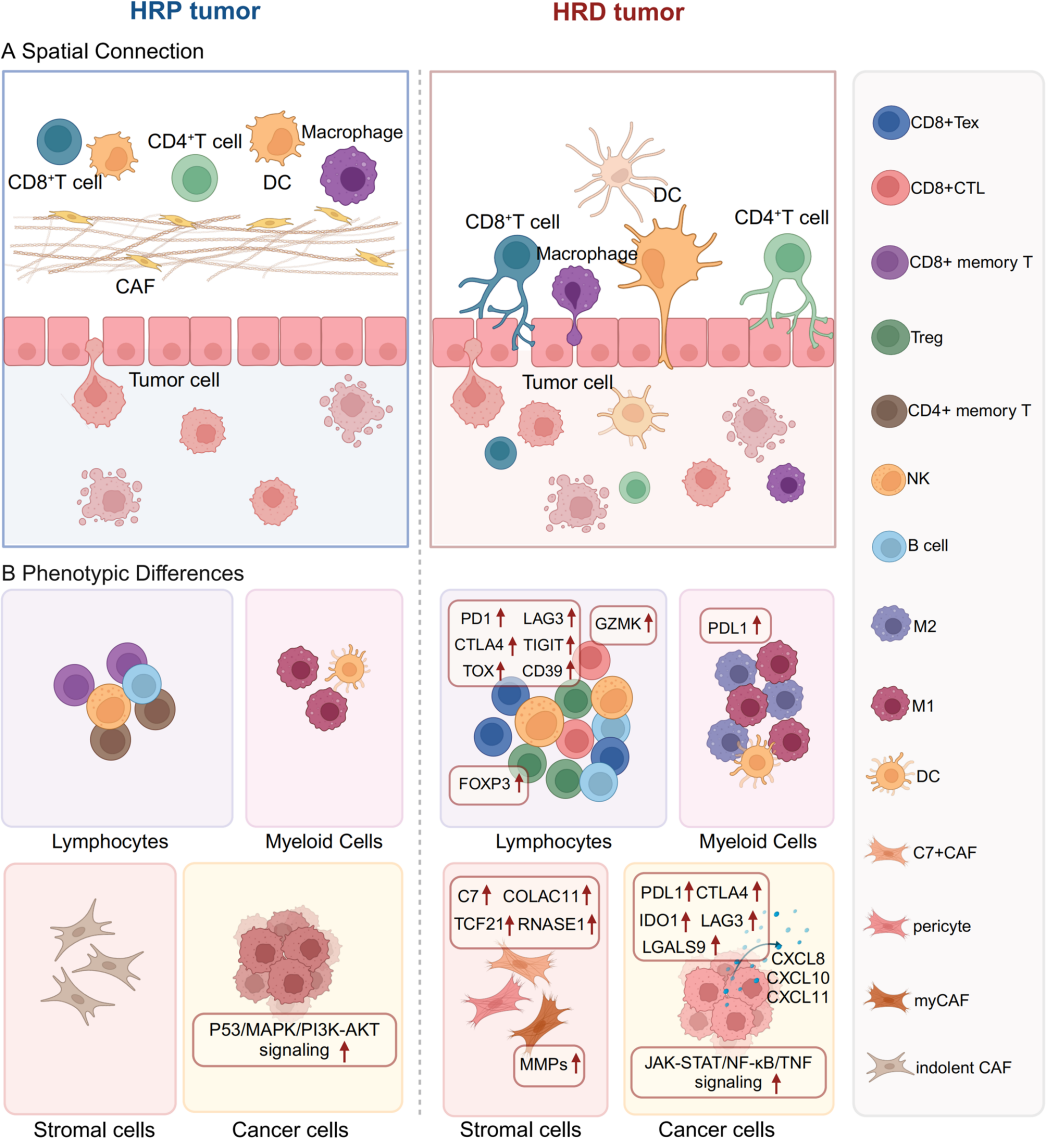
The distinctive phenotypes and spatial connection of cells in the TME of HRD cancers. (**A**) Spatial connection. In HRD tumors, CD4^+^/CD8^+^T cells, DCs, and macrophages are localized in closer proximity to tumor cells, showing strengthened spatial interactions with tumor epithelial cells, compared with HRP tumors. (**B**) Phenotypic differences of lymphocytes, myeloid cells, stromal cells, and cancer cells between HRD tumors and HRP tumors. HRD, homologous recombination deficiency; HRP, homologous recombination proficiency; TME, tumor microenvironment; Tex, exhausted T cell; CTL, Cytotoxic T lymphocyte; Treg, regulatory T cell; DC, dendritic cell; CAF, cancer-associated fibroblast

As previously mentioned, many types of cancer commonly harbor genomic alterations associated with HRD, including HGSOC, TNBC, pancreatic cancer, prostate cancer, NSCLC, and others [86]. HGSOC is an excellent tumor model of HRD because a majority of cases exhibit mutations or hypermethylation in BRCA1/2 or other important HRR genes [87]. Recently, several studies have demonstrated that HRD cancers present a unique TME with distinct phenotypes of immune cells and spatial tumor-immune interactions compared with homologous recombination proficient (HRP) tumors. These studies have shed light on the molecular, spatial, structural, and functional characteristics of this unique TME utilizing laboratory techniques, single-cell technologies, and multiplexed spatial analyses [38, 73]. Detailed elucidation on the interplay among diverse components in the TME of HRD cancers is conducive to characterizing a distinctive TME landscape for precise identification of therapeutic targets and prognostic assessment of HRD cancers. Therefore, we aim to describe the intricate interplay of tumor, immune and stromal cells, and the crosstalk among these cells and related cytokines (Fig. 2).

### T cells

T cells constitute a crucial component of the TME [88]. CD8^+^ T cells are potent effector cells that exert anti-tumor immune effects by specifically recognizing and eliminating cancer cells [89]. Additionally, CD4^+^ helper T (Th) cells exert influence on various immune cell subsets, with the Th1 and Th2 phenotypes playing dual roles in modulating anti-tumor immunity [90, 91]. Treg cells impede effective anti-tumor responses through different mechanisms, serving as gatekeepers of immune homeostasis [92]. Accumulating evidence has revealed that elevated T-cell density and activation signatures are associated with enhanced survival across diverse cancer types, suggesting the potential to utilize T-cell characteristics as prognostic indicators for oncology patients [93, 94].

Prior to the onset of tumors, the adaptive immune system exerts a role in the early phases. In the mucosal microenvironment of the fallopian tubes of patients with BRCA1 germline mutations, CD8^+^ T cells are conspicuously enriched in BRCA1 mutation carriers, and the proportion of clonal CD8^+^ T cells is significantly higher than that of the normal control group [95]. Similarly, the proportion of lymphocytes, particularly CD4^+^, CD8^+^, and NK/NKT cells, is markedly increased in the breast of donors carrying BRCA1/2 germline mutation. The most notable change is the enrichment of cytotoxic CD8^+^ T cells highly expressing perforin, granzyme B, IFN-γ, and TNF-α in the donor with BRCA1 germline mutation [96]. This implies that CD8^+^ T cells in the microenvironment of the fallopian tubes and the breast of BRCA1 mutation carriers have initiated the function of immune surveillance before tumorigenesis.

Additionally, several studies have demonstrated that there are significant differences in T cell phenotypes between HRP and HRD tumors in HGSOC patients, and there is also a higher diversity of T cell phenotypes [38, 45, 97]. Compared with wild-type (WT) tumors, significantly more adaptive immune response cells, especially tumor-infiltrating lymphocytes (TILs), are enriched in BRCA1/2 mutant TNBC, as well as prostate cancer and pancreatic cancer with BRCA2, PALB2, or ATM germline mutations. The HRD status is also correlated with the increased density of CD8^+^ and CD4^+^ T cells [42, 44, 98]. The infiltration of CD8^+^ T and CD4^+^ T cells is significantly increased in BRCA1/2 mutated HGSOC, and is positively correlated with the quantity of other immune-stimulating cells, indicating a coordinated recruitment phenomenon of immune cells in BRCA1/2 mutated tumors [38]. Notably, one study observed no statistically significant difference in T cell density or composition between the HRD tumor cohort and sporadic tumor cohort, whereas the level of T cell inflammation signature (TIS) was higher in HRD tumors than in sporadic tumors. HRD samples also possess a higher level of tissue-resident memory T cells (Trm), which is implicated in maintaining immunity. This indicates the existence of immune surveillance in the TME of HRD tumors, but the high level of Trm may also account for the potential cause of tumor immune evasion and progression [99, 100].

However, even though HRD cancers exhibit a CD8^+^ T cell-enriched state, these CD8^+^ T cells frequently manifest a dysfunctional phenotype. In the mucosal microenvironment of the fallopian tube or the breast, prior to HGSOC or breast cancer development, PD-1, LAG3, TOX, TIGIT, and CD39 positive exhausted T cells and NK/NKT cells are significantly enriched in BRCA1/2 mutation carriers, indicating that the early adaptive immune responses are initiated, whereas T cells undergo a transformation towards an immune exhaustion phenotype. This finding also suggests a possibility of a faster progression of HGSOC or breast cancer in BRCA1/2 germline mutation patients [95, 96]. Furthermore, the expression of molecules such as CD8, CD3, FOXP3, PD1, CTLA4, and GZMB is significantly increased in BRCA1 mutated tumor samples, signifying a robust T cell response in BRCA1 mutant TNBCs but a more prominent T cell exhaustion [42]. Meanwhile, dysfunctional CD8^+^ T effector cells characterized by high expression of cytotoxic markers (GZMK) and exhausted markers (PD-1, CTLA4, LAG3, and TIGIT) are significantly enriched in the peritumoral stroma and tumor tissues of HGSOC patients with HRD [45]. For HRP tumors, the CD8^+^ T cell phenotype is mainly concentrated on CD8^+^ central memory T (Tcm) cells and CD8^+^ effector memory T (Tem) cells [101]. This indicates that the HRD group has a greater potential for tumor-reactive T cells than the HRP group, and the interaction between T cells and antigens is significantly enhanced. The immune exhaustion phenotype of CD8^+^ T cells in HRD tumors is consistent with the dysfunction induced by chronic antigen exposure in solid tumors, and implies the considerable potential of immune checkpoint blockade (ICB) treatment for HRD tumors.

Furthermore, Treg cells are also enriched in the TME of HRD cancers. In HGSOC, prostate cancer, and pancreatic cancer cases with HRD phenotype, CD4^+^ Treg cells in the TME and circulating CD4^+^ Treg cells account for a higher proportion of T cells [44, 102, 103]. The quantities of CD3^+^CD8^+^GrB^+^, CD3^+^CD4^+^ cells, and FOXP3^+^ Treg cells are augmented in BRCA1-mutated TNBC when compared with BRCA-WT TNBC [104]. Additionally, there is a positive correlation between CD8^+^ T cell density and FOXP3^+^ T cell density in BRCA and ATM germline mutated cases. There is also a positive correlation between HRD score and FOXP3^+^ T cell density in the BRCA2/ATM germline alteration cohort [98]. Another study also demonstrated that the abundance of total Treg cells and effector Treg (eTreg) cells is significantly higher in HRD tumors. Moreover, eTreg cells and exhausted T (Tex) cells co-exist, and both display increased infiltration in HRD tumors [105]. In contrast, CD4^+^ Tcm cells undergo clonal expansion in the HRP group [101].

Collectively, in the TME of HRD cancers, CD8^+^ T cells recognize effective tumor antigen presentation signals and proliferate subsequently. However, while the infiltration of tumor-reactive T cells increases, immunosuppressive eTreg cells and Tex cells also co-exist, presenting a delicate balance between the states of anti-tumor and pro-tumor.

Moreover, the spatial interaction between T cells and tumor cells is also particularly distinctive in HRD cancers. In comparison with HR-WT tumors, the spatial composition of TME in BRCA1/2-mutated tumors is markedly different. In BRCA1/2-deficient tumors, CD4^+^/CD8^+^T cells, endothelial cells, and functional stromal cells show strengthened spatial interactions with tumor epithelial cells, and CD8^+^T cells are mainly distributed near the tumor cells [38]. In BRCA-deficient breast cancer and prostate cancer, CD4^+^ and CD8^+^ T cells exhibit a higher degree of infiltration into tumor tissues compared with the stroma [103, 106]. This phenomenon is attributed to the high expression of Ki67 in proliferating epithelial cells, which enhances interactions with immune cells and thereby improves immune surveillance. Spatially, this indicates more frequent communication between immune cells and tumor cells. In HR-WT tumors, however, the level of cell-cell interaction between tumor cells and CD4^+^/CD8^+^T cells is relatively diminished. Moreover, the ratio of CD4^+^ T cells and CD8^+^ T cells near proliferating tumor cells in BRCA1/2-deficient tumors is positively correlated with patient prognosis, suggesting that the coordination of immune surveillance through the spatial connection of cells improves the prognosis of patients [38]. In prostate cancer, there is an intriguing proposition regarding the clustered immune space (CIS) and free immune space (FIS). Namely, T cells mainly cluster in the stromal region in tumors, which is termed CIS, while the condition of T cells that are “free” and dispersed alone in the tumor area is defined as FIS. CIS and FIS reflect diverse levels of immune-tumor cell interactions. In comparison with WT tumors, HRD tumors have significantly more infiltrating free T cells, particularly CD8^+^ T cells, indicating that HRD tumors possess an FIS profile. CD8^+^ cells in the FIS are closer to the tumor cells than the clustered CD8^+^ cells in the CIS. The expression of the HLA-A, which is requisite for cytotoxic effector T cell immune recognition, is positively correlated with the proportion of free CD8^+^ T cells, suggesting that HRD tumors with an FIS profile-dominant TME have a higher level of immune surveillance [99].

However, PD-L1^+^ cancer cells are encompassed with a greater number of CD8^+^PD-1^+^ T cells within a short distance (radius < 30-µm) in HGSOC cases with HRD phenotype, compared with HRP tumors. The median distance between tumor cells and dysfunctional T cells is also shorter in HRD tumors than in HRP tumors. Before the development of breast cancer, PD-1^+^ immune cells in carriers with BRCA1 and BRCA2 germline mutations are preferentially localized near the epithelial tissue, which might be associated with early immune evasion in the malignant epithelial tissue [96]. Additionally, Treg cells are observed to be localized in close proximity to tumor cells in HGSOC and prostate cancer cases with HRD. These findings indicate the occurrence of frequent functional interactions between these cell populations, and serve as evidence in support of immune suppression [38, 103, 105].

### B cells

Despite the current immunotherapy research mainly focusing on T cells, growing evidence indicates that tumor-infiltrating B lymphocytes (TIL-Bs) also play a vital collaborative role in the TME [107, 108]. Exhausted or dysfunctional CD8^+^ and CD4^+^ TILs are found to express CXCL13 [109, 110]. This observation implies that, upon encountering the threat presented by tumor cells, exhausted or dysfunctional T cells might solicit support from B cells [111, 112]. This interaction between T and B cells eventually gives rise to a tertiary lymphoid structure, which actively participates in the adaptive immune response within the TME [113]. Therefore, it is held that TIL-Bs possess a positive prognostic value in most cancers [114].

In BRCA2-deficient breast cancer, activated B cells and immature B cells are enriched [42]. Compared with WT prostate cancer, the upregulated genes in the HRD phenotype cohort encompass B cell markers (CD79A, CD79B, MS4A1). The density of B cells in the stroma of gBRCA2-mutated samples is similar to that in the tumor region, and the higher density of B cells in the stroma of gBRCA2 samples might be the cause for the overexpression of B cell markers in the HRD cohort [99]. Pancreatic cancer cases with BRCA2/PALB2 germline alterations demonstrate a more enriched B cell population, including memory B cells and naïve B cells, and the abundance of B cells shows a positive correlation with the HRD score [44, 115].

### NK cells

Natural killer (NK) cells are the most important lymphocytes in the innate immune system and are regarded as the earliest immune effectors in the TME due to their potent immune surveillance function [116, 117]. A growing number of studies have indicated that the quantity, infiltration, and function of NK cells in the TME are positively correlated with the survival rate of cancer patients [118]. The number of NK cells is significantly associated with the long-term overall survival (OS) rate of melanoma patients [119]. Similarly, higher levels of tumor-infiltrating NK cells are associated with pathologic complete response and disease-free survival in breast cancer [120]. Furthermore, NK cells can drive immune cell infiltration and inflammation, and convert “cold” tumors into “hot” tumors, thereby promoting adaptive immunity and enhancing patient response to ICB [121].

Pancreatic cancer cases with BRCA2/PALB2 germline alterations exhibit a more enriched population of activated NK cells [44]. Similarly, compared with HRP cases with HGSOC, HRD cases exhibit higher expression of NK cell features, and the activated NK cells in HRD cancers imply better outcomes. The lower levels of NK cell infiltration are associated with a decreased survival rate in HRD patients with BRCA1 alterations [97].

As previously mentioned, in the breast samples of donors carrying BRCA1/2 germline alterations, the proportion of lymphocytes increases significantly, including GZMH^+^ NK and NKT cells. Meanwhile, the expression of LAG3, TIGIT, and TIM3 is augmented, suggesting the accumulation of immune exhaustion phenotypes of NK cells, which is consistent with the exhaustion phenotypes of T cells in the early stages of tumorigenesis [96].

### Macrophages and dendritic cells

Macrophages with anti-tumor effects are defined as M1 polarized macrophages expressing CD80, CD86, MHC-II, and iNOS. These macrophages retain the characteristics of APCs and participate in immune-active pathways, including chemokine signaling pathways, antigen processing, and presentation, as well as activation of adaptive immune cells and cytokine-cytokine receptor interactions [122–124]. Conversely, macrophages with pro-tumor effects are characterized by the expression of CD206, CD204, VEGF, CD163, and Arg-1, being defined as M2-type macrophages with immunosuppressive properties. M2 macrophages are marked by low expressions of MHC-II and inhibitory molecules such as PD-1, PD-L1, VISTA, B7-H4, and Tim3 [125–128]. Therefore, the unique features of tumor-associated macrophage (TAM) render it a potential therapeutic target [129–131]. Although the concept of M1 and M2 macrophages is no longer deemed appropriate, most studies continue to employ these related markers to characterize TAM attributed to a broad correlation between the expression of these markers and prognosis in tumor models and human cancers [132].

Despite the recognition that dendritic cells (DCs) lack direct anti-tumor activity, they nonetheless play a role in modulating anti-tumor immunity via their interactions with T cells. In other words, DCs play the “optimal supporting role” in anti-tumor immunity [133, 134]. A recent study has demonstrated that tumor T-cell inflammation is more closely correlated with the genetic characteristics of DCs, suggesting that antigen processing and presentation constitute a key step in immune recognition [135, 136].

In HGSOC, the infiltration rate of IBA1^+^ M2 and CXCL10^+^ M2 macrophages is markedly elevated in HRD tumors, while M1 macrophages are enriched in HRP tumors [38, 45]. Nevertheless, contradictory evidence demonstrates that a higher proportion of pro-inflammatory M1 macrophages is observed in HGSOC cases with HRD phenotypes. This might be attributed to more robust antigen-presenting interactions within the HRD group, which demands the recruitment of more immune cells and the establishment of an inflammatory environment. Meanwhile, HRP tumors are infiltrated with more inactive monocytes, and the deficiency of new antigen signals in the tumor leads to the polarization of immunosuppressive M2 macrophages [101]. Furthermore, in cases of TNBC with BRCA1 mutation and pancreatic cancer with BRCA2/PALB2 germline alterations, a more abundant macrophage population, encompassing M1 and M2 subtypes, is evident. This finding is in line with the conclusion from several previous studies on HGSOC that the number of M1 or M2 type macrophages in HRD tumors increases independently [38, 44]. These contradictory results might be associated with the HRD score of the tumors. It is demonstrated that M1 macrophages predominate in tumors with higher HRD scores, whereas M1 and M2 macrophages seem to have an equal presence in tumors with intermediate HRD scores [115].

Additionally, the PD-L1 expression on macrophages increases in samples from gBRCA1/2 mutated breast cancer, suggesting the formation of an immunosuppressive environment locally prior to the tumorigenesis [96]. Similarly, both types of TAM in HGSOC with HRD phenotype and BRCA1 mutated TNBC also exhibit higher expression of PD-L1 [45, 104]. As the HRD score rises, the signal crosstalk between macrophage-related APOB-TREM2 and CD52-SIGLEC10 is concomitantly augmented, which is related to tumor immune evasion [115, 137].

In addition, the spatial interaction between TAMs and other cells in HRD cancers exhibits distinct characteristics. In BRCA1/2-mutant HGSOC and BRCA2/ATM-mutant prostate cancer, M2 macrophages exhibit a preferential accumulation within regions of proliferating tumor cells. Conversely, in HR-WT tumors, they are primarily present in the stroma region [38, 103]. A notable increase is also observed in the localization of M1 macrophages in BRCA1 mutant samples, with these cells infiltrating into the tumor cells, while no such phenomenon is observed in HRP samples [45]. Similar to PD-L1^+^ cancer cells, PD-L1^+^ macrophages are enriched with a greater number of CD8^+^ PD-1^+^ T cells within a short distance, which is particularly common in BRCA2-mutated HGSOC cases but absent in HRP tumors. These findings support the hypothesis that the upregulation of PD-L1 in tumor cells and myeloid cells serves as a negative feedback mechanism to suppress T cell activation in HRD cancers [45].

### Cancer-associated fibroblasts

Cancer-associated fibroblasts (CAFs) constitute a key component of the tumor stroma. Within the TME, CAFs mediate communication between tumor cells and other immune cells via the diverse cytokines and exosomes they produce, not only facilitating tumor proliferation but also inducing immune evasion [138–140]. Moreover, as a crucial component of stromal cells, CAFs can induce the degradation of the extracellular matrix (ECM) by releasing matrix metalloproteinases (MMPs) and synthesizing new ECM proteins, providing structural support for tumor invasion and angiogenesis, which largely contributes to drug resistance in cancer treatment [141–143].

BRCA1 alteration carriers exhibit a significant enrichment of fibroblasts expressing EMT-related genes. These subpopulations highly express CXCL12 and IL-6 genes, which are known to regulate inflammatory responses and activate the JAK/STAT signaling pathway in HGSOC [144]. This is in line with the high expression of EMT-related genes in the epithelial cells of the fallopian tubes of BRCA1 alteration carriers, suggesting the existence of special interactions between CAFs and epithelial cells that promote the initiation of HGSOC [95].

The pancreatic ecosystem shows high levels of infiltrating fibroblasts in the HRD group, including antigen-presentation-associated fibroblasts (apCAF) and myofibroblastic CAFs (myCAF) [115]. Moreover, HRD cancers demonstrate a high degree of infiltration of C7 + CAFs (C7, COLAC11, TCF21, and RNASE1), meso-fibroblasts, pericytes, and myCAFs, which actively engage in inflammatory responses, angiogenesis, and responses to type I/II interferons (IFNs). Among these, myCAFs show a high expression level of MMPs, indicating their potential role in degrading the ECM to facilitate tumor cell invasion and metastasis, while providing migration routes for tumor cells to invade the blood and lymphatic systems. Additionally, a high level of PD-L1 expression is detected in the tumor stroma in BRCA1-mutated TNBC, indicating the existence of immune exhaustion in the HRD tumor stroma [43]. In contrast, HRP tumors display a significant infiltration of “indolent CAFs” with limited functionality and minimal interaction with other cell subtypes, namely ADH1B^+^ CAFs and MYH11^+^ SMCs, suggesting the formation of an immune exclusion barrier [101].

### Cancer cells

Three immune signaling pathways, including JAK-STAT, NF-κB, and TNF signaling pathways, are conspicuously augmented in the primary tumor sites of HRD cancers, which might imply the origin of the immune signal of HRD tumor cells. In comparison with the primary tumors of HRP, MHC-I/II genes are highly expressed in HRD cancers [45, 105]. Moreover, epithelial cells of HRD tumors are profoundly engaged in the antigen presentation process and the differentiation of Th1, Th2, and Th17 cells [101]. Furthermore, epithelial cells of HRD tumors could excrete a variety of immune chemokines such as CXCL8, CXCL10, and CXCL11 to recruit anti-tumor immune cells, suggesting an active immune response in the TME of HRD cancers [45]. In contrast, epithelial cells of HRP tumors exhibit a close association with cancer-related pathways, including the cell cycle, P53 signaling pathway, MAPK signaling pathway, and PI3K-AKT signaling pathway [101]. These results further validate the active immune interaction between tumor cells and immune cells and the initiation of multiple immune pathways in the TME of HRD cancers.

However, in cancer cells with IFN response, the expression of many co-inhibitory molecules is significantly increased, including PD-L1, Galectin-, indoleamine 2,3-dioxygenase (IDO), and ICOSL. The upregulation of these molecules facilitates the immune suppression function of Tregs/Texs [105]. In both HGSOC with HRD phenotype and BRCA1 mutant TNBC, CD274 (PD-L1) expression on tumor cells is also upregulated [43, 45]. Additionally, BRCA1/2-deficient breast cancers have a higher expression of immunosuppressive molecules compared with BRCA1-WT breast cancers in WSI and TCGA cohorts, including CTLA-4, IDO1, and LAG3 [145]. The expression of the LGALS9 gene is significantly upregulated in HRD tumor cells, which encodes a protein that is a ligand for HAVCR2/TIM3, a classical immunosuppressive molecule [146, 147]. These findings suggest that the TME of HRD cancers exhibits both active immune responses and immune-suppressive signals, which aligns with previous observations in immune cell populations.

## The distinctive features of molecular mechanism in the TME of HRD cancers

**Fig. 3:**
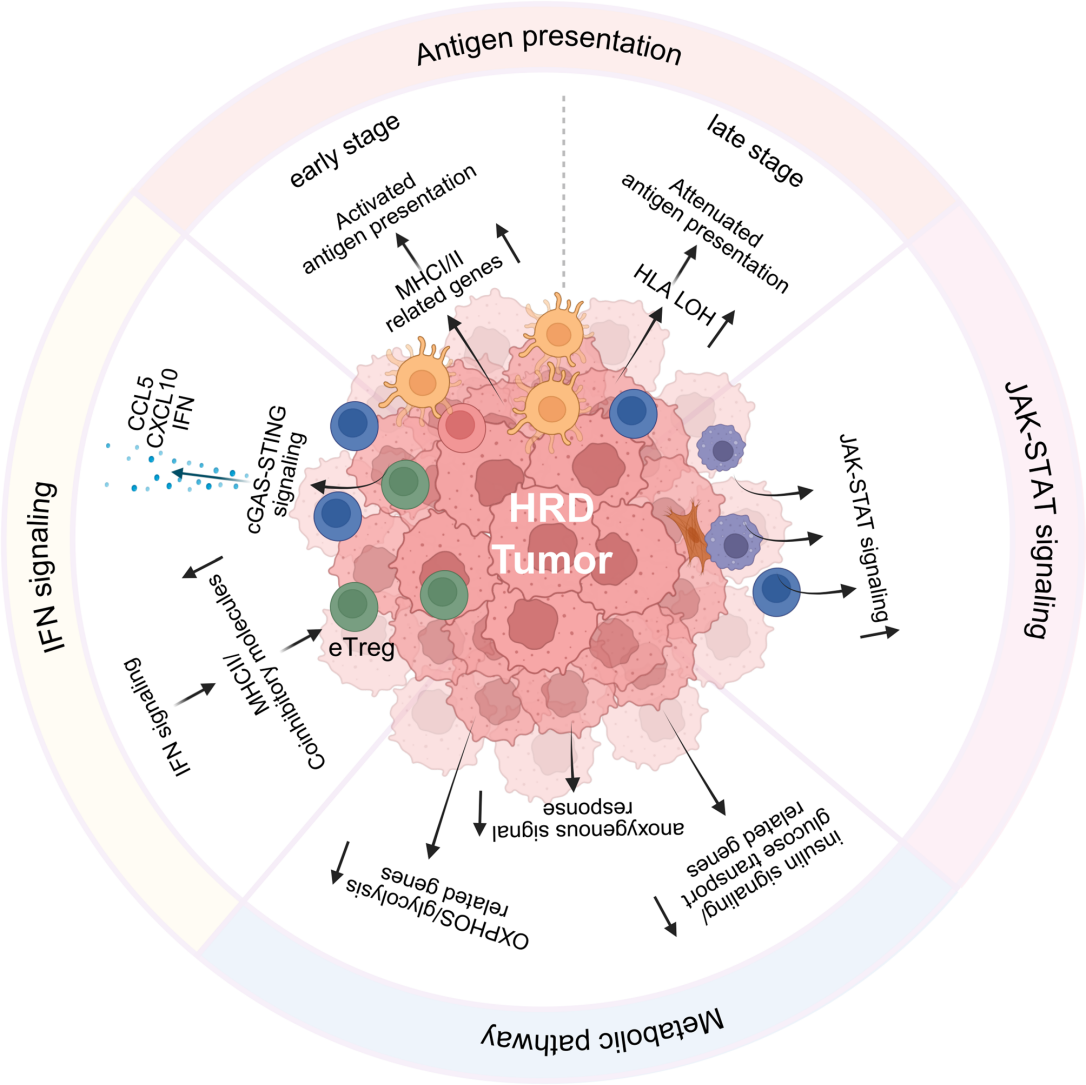
The distinctive features of molecular mechanism in the TME of HRD cancers. The TME of HRD cancers exhibits distinct characteristics in cellular interplay, signaling pathways, and other aspects. These features are reflected in the differential states of antigen presentation pathways during early and late stages, the upregulation of JAK-STAT and IFN signaling and the significant increase in oxygen demand by cells in the TME. TME, tumor microenvironment; HRD, homologous recombination deficiency

As previously discussed, the TME of HRD cancers displays a distinct cellular phenotypic landscape within a spatial framework. More evidence further indicates that the TME of HRD cancers is characterized by molecular alterations across four critical pathways: (1) dynamic regulation of antigen-presenting capacity, (2) upregulation of IFN signaling, (3) activation of the JAK-STAT pathway, and (4) metabolic reprogramming centered on energy utilization (Fig. 3). These pathways interweave with immune cells, enabling initial immune engagement while simultaneously orchestrating progressive immune suppression. Consequently, these interplay between immune cells and molecular mechanisms shape the distinctive TME landscape observed in HRD cancers.

### Antigen presentation

Antigen presentation serves as a fundamental process of the adaptive immune response, which establishes a connection between immune cells and tumor cells via MHC class I/II. It is discovered that the antigen presentation capacity of tumor cells or professional APCs is not invariable throughout the development of HRD cancers. The absence or mutation of HLA genes significantly influences the anti-tumor properties of T cells.

In HRD tumors at the early stage or during sensitive treatment periods, the process of antigen presentation is more vigorous. Prior to the progression to breast cancer, the expression of the HLA-DQB1 gene in BRCA2-mutated breast cells has been identified to be significantly upregulated, which is correlated with MHC class II and plays a critical role in antigen presentation [96]. Additionally, the gene with the most pronounced differential expression within the HRD cohort of prostate cancer is HLA-A, which is indispensable for the immune recognition of cytotoxic effector T cells [99]. Among PACCs with germline HR alterations, only 14% are detected with LOH of HLA class I [44]. In HGSOC patients with chemosensitivity where BRCA1/2 mutants are predominant, HLA-DMB, TAP1, TAP2, and TAPBP genes on the 6th chromosome are also upregulated [148]. These genes are all associated with antigen presentation. These results indicate that the antigen presentation process in early-stage HRD cancers is more active, and the capacity of immune cells to recognize tumors is augmented, which is aligned with the previous discoveries of increased immune cell infiltration and enhanced immune surveillance phenotypes.

However, during the disease progression, antigen presentation is gradually attenuated. In lethal metastatic breast cancer, patients with ATM alterations exhibit more clonal HLA LOH, while those with BRCA2 alterations present more subclonal HLA LOH [73]. In patients with advanced HGSOC, HRD cancers manifest more HLA class I gene subclonal LOH and LOH, which is a reported immune evasion mechanism in lung cancer and other solid tumors [149]. A considerable proportion of clonal 6p LOH is observed in patients with BRCA1/2 alterations, and the functional consequence of 6P LOH in HRD cancers, namely, an increase in dysfunctional CD4^+^ and CD8^+^ T cells, is also demonstrated [45]. In conclusion, the association between the loss and mutation of relevant genes and T-cell dysfunction implies that the gradually emerging immune exhaustion during the progression of HRD cancers might be related to the evolutionary selection of antigen presentation capacity.

### IFN signaling

Plenty of evidence suggests that the IFN signaling is upregulated in HRD cancers. The CXCL10/CXCR3 axis has been reported as a crucial factor for regulating the chemotaxis of CD4^+^ and CD8^+^ T lymphocytes [150]. The cGAS-STING pathway is activated in BRCA1-deficient breast cancer cells, leading to an upregulation of the expressions of CCL5 and CXCL10 and promotion of lymphocyte migration [106]. In pancreatic cancer, cases with BRCA2/PALB2 germline alterations display higher IFN responses, encompassing both type I (α/β) and type II (γ) [44]. In the HRD cohort of prostate cancer, the upregulated genes also encompass IFN-related cytokines and chemokines, including CXCL13, CXCL9, and CXCL10 [99]. In HGSOC, BRCA1 deficiency elicits type I IFN secretion and STING-mediated intrinsic inflammation, which promotes T lymphocyte infiltration by enhancing the expressions of CCL5 and CXCL9 [151, 152]. In HRD HGSOC patients at the end stage, most samples exhibit hypermethylation of CCL5, which not only affects the function of CD8^+^ T lymphocytes but also promotes the differentiation of M2 macrophages, leading to adaptive immune suppression and poor prognosis [73, 153].

Although IFN signaling has been conventionally regarded as a vital stimulator of anti-tumor immunity, it also plays a central role in immune evasion within tumors [154, 155]. On the one hand, the upregulated IFN signal in HRD cancers fosters a positive response to anti-tumor immunity. On the other hand, it gives rise to the emergence of an immunosuppressive Treg phenotype through an extensive immune editing with the upregulation of MHC II and coinhibitory molecules, attaining a subtle balance within the TME in cases when tumor-responsive effector T cells surge [105].

### JAK-STAT pathway

The JAK-STAT pathway can be activated by various cytokines, among which type I/II IFNs are the most crucial upstream signaling molecules [156]. In HRD cancers, the JAK-STAT pathway is upregulated in tumor cells, CD4^+^/CD8^+^ T cells, and macrophages, implying a common upstream effector in this pathway within HRD cancers. Moreover, given the robust positive correlation between the IFN signaling in DCs and the activation of the JAK-STAT pathway in cancer cells, T cells, and macrophages, the increased IFN activity in HRD cancers might serve as an activator of the JAK-STAT pathway and is accompanied by the upregulation of HLA gene and immune co-inhibitory molecules such as PD-L1 [45, 105]. As mentioned above, HGSOC with HRD is enriched with CXCL10^+^ macrophages. Since CXCL10 is a target of the JAK-STAT pathway, macrophages in HRD demonstrate higher JAK-STAT pathway activity [45]. Therefore, the activation of the JAK-STAT pathway is in accordance with the upregulation of the IFN signal in HRD cancers.

### Metabolism pathway

Prior to tumor progression, the fallopian tube epithelial cells of BRCA1 mutation carriers upregulate genes related to oxidative phosphorylation (OXPHOS) and glycolysis, such as UQCRB and LDHB [95], compared with the WT population, indicating that BRCA1-deficient cells have considerably higher energy requirements than normal cells in the extremely early stage of HGSOC. OXPHOS and glucose metabolic pathways are also upregulated in HGSOC autopsy samples. The increase in expression of the OXPHOS pathway is positively correlated with HRD score, and high-OXPHOS tumors appear to be more sensitive to chemotherapy [157].

In breast cancer, the anoxygenous signal response is markedly upregulated in HRD samples when compared with HRP samples [158]. Intriguingly, genes associated with insulin signaling and glucose transport, including IRS1, IRS2, CACNA1D, SOCS3, and PRKCZ, are also upregulated in HRD samples [78], which is in line with the findings in HGSOC [73]. In summary, in comparison with HRP cancers, HRD cancers exhibit a greater reliance on energy metabolism to fulfill the cellular growth requirements, potentially offering a promising therapeutic opportunity.

## The clinical significance of alterations in the HRD genes and the unique TME of HRD cancers

### Biomarker of prognosis

In comparison with short-term survivors, HGSOC long-term survivors and intermediate survivors possess higher germline and somatic HR-gene alteration rates. These alterations encompass germline alterations in BRCA1, BRCA2, BRIP1, PALB2, and RAD51C, somatic alterations in BRCA1, BRCA2, ATM, CDK12, PTEN, RAD51B, RAD51C, and RAD51D, and promoter methylation of BRCA1 and RAD51C. RB1 holds an atypical function in HR, and mutations in both RB1 and BRCA1 are associated with better prognosis. The concurrent mutation frequency of three or more HR-genes is also higher among long-term survivors, and this character is significantly correlated with favorable prognosis [97].

In HGSOC, CCNE1 amplification and BRCA1 mutation are mutually exclusive. Notably, CCNE1 amplification is significantly more prevalent in patients with short-term survival, serving as a poor prognostic indicator for primary platinum-resistant disease [97]. Tumors with combined deficiencies of BRCA1 and BRCA2 might be more susceptible to platinum-based chemotherapy. The BRCA1-mutated group has the longest PFS, suggesting that these tumors are particularly sensitive to platinum-based drugs [97]. In breast cancer, multiple gene defects in DNA repair could mitigate tumor resistance to chemotherapy [97, 159].

Thirteen core genes in the HR pathway, including BARD1, BLM, BRCA1, BRCA2, BRIP1, MRE11, NBN, PALB2, RAD50, RAD51, RAD52, TP53BP1, and XRCC2, are identified to be mutated in patients undergoing neoadjuvant immunotherapy. Notably, these thirteen genes are significantly enriched in patients who achieve a major pathologic response (MPR). Regardless of the treatment regimen and clinicopathological characteristics, the frequency of HR pathway alterations is higher in patients with better responses to immunotherapies. Moreover, in patients receiving immunotherapies, mutations in the HR pathway correlate with higher TMB and longer survival, substantiating the potential of HRD as a novel prognostic biomarker for immunotherapy [160].

In addition to alterations in the HRD genes, the tumor immune microenvironment composition demonstrated profound prognostic implications as well. In HGSOC patients with BRCA1/2 deficiency, the high infiltration of CD4^+^T cells is significantly correlated with a longer Platinum-Free Interval (PFI), which might be an independent predictive indicator. The high infiltration of CD8^+^T cells is associated with the improvement of PFI regardless of BRCA deficiencies [38]. Another study also shows that HGSOC patients exhibiting higher densities of immune cells achieve markedly prolonged OS and a favorable PFS. Notably, multivariable Cox regression reveals that CD8^+^, CD20^+^, and CD103^+^ emerge as positive prognostic biomarkers in the adjusted analysis [161].

Furthermore, CIBERSORTx-based immune cell profiling of HGSOC bulk RNA-seq data reveals significant associations between immune cell composition and clinical outcomes [97]. Specifically, higher proportions of activated memory CD4^+^ T cells and plasma cells correlate with better OS, while elevated levels of resting mast cells and M2 macrophages are linked to poorer outcomes. Patients with the longest PFS and OS show enrichment of plasma cells, activated memory CD4^+^ T cells, M1 macrophages, and resting NK cells. Those with intermediate survival exhibit increased CD8^+^ T cells, activated NK cells, Treg cells, and follicular Th cells. Conversely, tumors from patients with the worst outcomes and lowest neoantigen burden are predominantly enriched for resting mast cells and DCs. Besides, univariate analysis identifies 7 OS-associated features, among which activated CD4 memory T cells and plasma cells are shown to be two individually protective factors. When combined in a multivariable regression model, plasma cells remains an independent prognostic predictor [97].

In TNBC patients, HRD is associated with favorable patient response to anthracycline, cyclophosphamide and taxane (ACT) chemotherapy and improved patient prognosis, including prolonged ACT chemotherapy failure-free interval (FFI), OS and disease-specific survival (DSS). However, it is notable that a subtype of TNBC patients with features of ACT-sensitive and HRP is identified to exhibit heightened TILs, IFN-γ signaling, NK cell abundance, and immune checkpoint markers, such as PD-L1, CTLA-4 and IDO1, suggesting potential responsiveness to ICB [162].

Among patients with prostate cancer undergoing immunotherapies, HRD patients demonstrate a more pronounced response to PSA50. HRD patients have a higher overall response rate (ORR) and exhibit a trend towards a superior 12-month PFS [79]. Additionally, the FIS of T cells in prostate tumors mentioned previously could also serve as a characteristic indicator of immunotherapies. Smaller tumor volume, lower Gleason score, and longer time intervals between treatment and recurrence or metastasis all show a positive correlation with FIS levels, as CD8^+^ cells are more prone to approach tumor cells, leading to better disease control [99].

### Therapeutic potential of immunotherapy in HRD tumors

#### Advances in preclinical models

In several preclinical models, combination therapy involving the targeting of immunosuppressive factors within the TME alongside PARPi has demonstrated promising advancements (Table 1). As Treg cells that play immunosuppressive and pro-tumorigenic roles are enriched in the TME of HRD cancers, selective Treg cell-depletion is a feasible strategy for HRD cancer treatment. CCR8 is a marker identified recently to predominantly be expressed on eTreg cells, the principal subset of Treg cells that exert immunosuppressive effects within the TME [163]. In the *Trp53*^-/*-*^*Brca*^-/-^ ID8 mouse model, administration of anti-CCR8 humanized therapeutic monoclonal antibody (mAb) remarkably reduces the tumor burden [105]. Notably, the anti-tumor efficacy of combining PARPi with the anti-CCR8 mAb is significantly superior to that of either agent alone. Similar results are observed in the *Brca2*^-/-^ EO771 mouse model as well. Besides, the combination of PARPi with anti-CD25 mAb, another commonly used Treg cell depletion therapy, exhibits a likewise potent anti-tumor effect [105]. These findings underscore the therapeutic potential of Treg-cell-targeted therapeutics and their synergistic effect with PARPi in HRD cancers.

**Table 1:** Advances of immunotherapy in HRD tumors in preclinical models

Cancer type	Immunotherapy	Target	Combined treatment	Model	Ref
OC	Anti-CCR8 mAb	Treg cells	Niraparib	*Trp53*^*−/−*^*Brca1*^*−/−*^ ID8	[105]
BC	Anti-CCR8 mAb	Treg cells	Niraparib	*Brca2*^*−/−*^ EO771	[105]
BC	Anti-CD25 mAb	Treg cells	Niraparib	*Brca1*^*−/−*^ EO771	[105]
PDAC	Anti-CD52 mAb	Macrophages	Olaparib	PAN02	[115]
OC	Anti-CD47 mAb	Macrophages	Niraparib	*Brca1*^*−/−*^ SKOV3	[165]
OC	TAX2	Macrophages	Olaparib	*Trp53*^*−/−*^*Brca*^*−/−*^ ID8	[170]
OC	CCL5 neutralizing antibody	CAF	Olaparib	BRCA1/2-mutant SKOV3 xenografts	[171]
OC	Bepotastine	CAF	Olaparib	HRD-positive PDX	[172]
BC	STING agonist	cGAS-STING	Olaparib	*Trp53*^*−/−*^*Brca1*^*−/−*^ GEMM	[173]

Abbreviation: OC, ovarian cancer; BC, breast cancer; PDAC, pancreatic ductal adenocarcinoma

A recent study analyzes the TME of PDAC tumors with different HRD statuses through high-dimensional multi-omic spatial profiling and figures out the therapeutic significance of CD52 in HRD tumors [115]. CD52 is previously demonstrated to act as a suppressor of T-cell activation and proliferation through interacting with SIGLEC10 [164]. In this study, however, the CD52-SIGLEC10 axis is identified as macrophage-related signaling, and its activity increases with rising HRD scores. Treatment with anti-CD52 could induce CD68^+^ M1-like macrophage expression, promote tumor regression, and inhibit TGF-β1 signaling and epithelial-mesenchymal transition (EMT). Intriguingly, macrophage induction and tumor suppression are enhanced when PARPi and anti-CD52 are used in combination, indicating a novel combined pharmacotherapy [115].

CD47 is another target associated with macrophages for immunotherapies in HRD cancers. CD47 is widely accepted as a macrophage immune checkpoint that interacts with the ligand signal regulatory protein α (SIRP-α) on macrophages to mediate a “don’t eat me” anti-phagocytic signal, and is implicated in the immunosuppressive TME [165]. Notably, it is discovered that PARPi could induce the upregulation of CD47 expression [165]. This finding provides a rationale for the combination of anti-CD47 therapy and PARPi. Indeed, both anti-CD47 monotherapy and the combined treatment of anti-CD47 with PARPi facilitate macrophage-mediated in-vitro phagocytosis, boost STING signaling, and eliminate tumor growth in BRCA-deficient ovarian cancer models [165]. Besides, the CD47/TSP-1 axis is also gaining much attention for its promoting effects on tumor survival and cancer stem cell differentiation, and inhibitory effects on anti-tumor immunity and therapy sensitivity [166–169]. TAX2 is a CD47-derived peptide that could selectively blockade the binding between TSP-1 and CD47. Sequential treatment with PARPi and TAX2 markedly retards tumor progression and prolongs survival in the *Trp53*^-/-^*Brca*^-/-^ ID8 mouse model, and TAX2 is efficacious regardless of PARPi sensitivity [170].

In addition to Treg cells and macrophages, CAF is another manageable component within the TME of HRD cancers. We previously discovered that upon PARPi treatment, stromal CAFs could be activated by autocrine CCL5 through the NF-κB signaling pathway in ovarian cancer [171]. The presence of these CAFs partially contributes to tumor resistance to PARPi. Accordingly, in both BRCA1/2-mutant and BRCA1/2-WT xenograft models, treatment with CCL5 neutralizing antibodies significantly inhibits PARPi-induced CAFs and decreases tumor volumes. Better efficacy is observed when PAPRi and anti-CCL5 antibodies are combined, which offers a conceptual strategy of targeting CCL5 to overcome PARPi resistance [171]. Furthermore, we find that PARPi could induce CAFs to exhibit a senescence-associated secretory phenotype (SASP) in a p21-dependent manner, which in turn confers PARPi resistance to ovarian cancer [172]. Importantly, utilizing the GLAD4U database, we screen out an H1-antihistamine, bepotastine, which exerts a reversal effect on PARPi-triggered SASP in CAFs via suppressing the NF-κB signaling pathway. In HRD-positive patient-derived xenografts, bepotastine improves tumor sensitivity to PARPi and displays a favorable anti-tumor efficacy [172].

Despite the ability of HRD cancers and PARPi per se to induce aberrantly damaged DNA accumulation to activate the cGAS-STING pathway, it is demonstrated that the combination of PARPi and STING agonist could trigger more robust STING activation, leading to reinforced downstream IFN signaling and T-cell and DC functions [173]. In the K14-Cre-*Brca1*^f/f^ *Trp53*^f/f^ TNBC model, the combination of PARPi and STING agonist not only clears tumors and promotes mouse survival, but also contributes to the development of immunologic memory, the latter of which is validated via re-implanting tumors into mice with complete tumor clearance [173].

Moreover, evidence suggests that PARPi sensitized BRCA mutant prostate carcinoma cells to NK cell-killing and antibody-dependent cellular cytotoxicity (ADCC) in a death receptor TRAIL-R2 dependent manner, indicating the promise of the combination of PARPi and NK- or ADCC-utilized therapy [174].

#### Advances in clinical trials

At present, the most commonly evaluated immunotherapies in clinical trials for HRD cancers are immune checkpoint inhibitors (ICIs), including anti-PD-1/PD-L1 and CTLA-4 antibodies (Table 2). Notably, various strands of evidence suggest that PD-L1 is upregulated in HRD tumors. On the one hand, DSB is involved in PD-L1 regulation. Upon DSB, ATM/ATR/Chk1 kinases are activated, leading to the PD-L1 upregulation, among which the Chk1 activation is the core step [175]. Moreover, the production of abundant dsDNA boosts type I IFN signaling through the cGAS-STING pathway, and subsequently activates JAK1/JAK2-STAT1/STAT2/STAT3-IRF1 axis. IRF1 binds to the promoter of PD-L1 and enhances its expression [176]. On the other hand, the application of PARPi in HRD cancers could induce PD-L1 upregulation by suppressing GSK3β [177]. These findings provide a solid theoretical basis and promising therapeutic potential for the adoption of ICIs in HRD cancers and their combination with PARPi.

**Table 2:** Advances of immunotherapy in HRD tumors in clinical trials

Cancer type	Immunotherapy	Target	Combined treatment	Endpoint outcomes	Trial ID	Phase	Ref
Advanced tumors with HRD	Tislelizumab	PD-1	Pamiparib	/	NCT04985721	2	
HRD^+^/HR^+^/​HER2^−^ Advanced BC	Adebrelimab	PD-L1	Fluzoparib	/	NCT06254066	2	
HRD^+^/ HER2^−^ Advanced BC	Camrelizumab	PD-1	Fluzoparib	/	NCT05656131/ NCT05085626	2	
HRD^+^ Solid Tumors	‌Dostarlimab	PD-1	Niraparib	/	NCT04983745	2	
HRD^+^ advanced CRC	Pembrolizumab	PD-1	Olaparib	/	NCT05201612	2	
HRD^+^SCLC	Durvalumab	PD-L1	Olaparib		NCT06419179	2	
Pleural mesothelioma and NSCLC harboring HRR mutations	Dostarlimab	PD-1	Niraparib	ORR: 6% (95% CI: 0.1–28.7) mPFS: 3.1 (95% CI: 2.7-NA) mOS: 4.2 (95% CI: 1.58-NA)	NCT04940637	2	[186]
HRD^+^Cholangiocarcinoma	‌Dostarlimab	PD-1	Niraparib	/	NCT04895046	2	
HRD^+^/ER^+^metastatic BC	Atezolizumab	PD-L1	Pabociclib/ Endocrine/ Talazoparib	/	NCT04819243	2	
HRD^+^OC	Pembrolizumab	PD-1	Olaparib	ORR: 70% (95% CI, 45.7–88.1) 12-month PFS: 91.7% (95%CI, 53.9–98.8)	NCT04417192	2	[187]
HRD^+^metastatic PDAC	Pembrolizumab	PD-1	Olaparib	ORR: 8% DCR: 62% (95% CI: 41–80) mPFS: 4 (95% CI, 2.1–5.4) mOS: 14 (95% CI, 10-NA)	NCT04666740	2	[188]
HRD^+/−^ metastatic gastric and GEJ cancers	Pembrolizumab	PD-1	Olaparib/ Stereotactic Body Radiation Therapy	/	NCT05379972	2	
Platinum-resistant relapsed/metastatic ovarian cancer with HRR gene mutation	Adebrelimab	PD-L1	Fluzoparib/ Non-platinum chemotherapy	/	NCT06600841	2	
HRD^+^platinum-resistant recurrent OC	Durvalumab/ Tremelimumab	PD-L1/ CTLA-4	Olaparib/ Cediranib/ Chemotherapy	ORR: 27.6% (95% CI: 16.7–40.9) DCR: 60.3% (95% CI: 46.6–73.0) 6-month PFS: 30.88% (95% CI: 19.15–43.36) 6-month OS: 79.53% (95% CI: 65.11–88.50)	NCT03699449	2	[189]
Advanced pancreatic cancer with HRD	Epacadostat/ Pembrolizumab	IDO1/PD-1	/	/	NCT03432676	2	
HRRm/HRD^+^ advanced	Pembrolizumab	PD-1	Olaparib	/	NCT04123366	2	[190]
HRD^+^ TNBC	Durvalumab	PD-L1	Olaparib	/	NCT05209529	2	
Unresectable or metastatic HER2^−^/HRD^+^ BC	Pembrolizumab	PD-1	Olaparib	/	NCT05033756	2	
Advanced CRC with HRD or microsatellite instability	‌Dostarlimab	PD-1	Niraparib	/	NCT06365970	2	
BC with DDR gene mutation	HX008	PD-1	Niraparib/ Trastuzumab/ Pyrrolitinib	ORR: 79% DCR: 96% mPFS: 7.4 (95% CI: 5.4–12)	NCT04508803	2	[191]
Prostate cancer with HRD	Vudalimab	PD-L1/ CTLA-4	Olaparib/ Docetaxel / Cabazitaxel	/	NCT05005728	2	[192]
Recurrent gliomas with HRD	Pembrolizumab	PD-1	Olaparib/ Temozolomide		NCT05188508	2	[193]
Metastatic pancreatic cancer with DDR gene mutation	Durvalumab	PD-L1	Olaparib	/	NCT05659914	2	[194]
Advanced solid tumors with HRD	Pembrolizumab	PD-1	IDE-161	/	NCT05787587	1	
Platinum-resistant EOC, EC, PPC or cervical cancers with HRD	Nivolumab	PD-1	Debio 1143	ORR: 2.9% mPFS: 1.9 (95% CI: 1.7–2.7) mOS: (95% CI: 6.0-15.9)	NCT04122625	2	[195]
Advanced OC with HRD	Durvalumab	PD-L1	Olaparib/ Bevacizumab/ Carboplatin + Paclitaxel	/	NCT03737643	3	[196]
Newly diagnosed OC	Nivolumab	PD-1	Rucaparib	/	NCT03522246	3	[197]
gBRCAm HER2^−^ metastatic BC/ gBRCAm OC	Durvalumab	PD-L1	Olaparib	28-wk DCR: 65.6% (90% CI: 49.6–79.4) ORR: 71.9% (95% CI: 53.25–86.25) mPFS: 11.1 (95% CI: 8.2–15.9)	NCT02734004	1/2	[178, 198]
Advanced BRCA-mutated BC	Pembrolizumab	PD-1	Olaparib	/	NCT03025035	2	
Prostate Cancer with BRCA2 inactivation or BRCAness signature	Ipilimumab/ Nivolumab	CTLA-4/PD-1	/	ORR: 38% (95% CI: 22–55) mPFS: 4.0 (95% CI: 3.5–12.0)	NCT04717154	2	[199]
Prostate Cancer with HRD	Nivolumab	PD-1	/	/	NCT03040791	2	
Prostate Cancer with HRD	Pembrolizumab	PD-1	Chemotherapy	/	NCT03248570	2	
Solid tumors with a BRCA or ATM defect	Avelumab	PD-L1	Talazoparib	ORR: 26.4% (BRCA1/2 cohort) 4.9% (ATM cohort)	NCT03565991	2	[182, 200]
TNBC or OC	Pembrolizumab	PD-1	Niraparib	ORR: 18% (90% CI: 11–29) DCR: 65% (90% CI: 54–75)	NCT02657889	1/2	[183, 201, 202]
BRCA mutant non-HER2-positive BC	Atezolizumab	PD-L1	Olaparib	PFS: 7.0 (95% CI: 5.5–11.5) (O arm) 7.67 (95% CI: 5.6–10) (O + A arm); mOS: 26.5 (95% CI: 19.2- NR) (O arm) 22.4 (95% CI 16.6–31.3) (O + A arm)	NCT02849496	2	[185]
Locally advanced or metastatic solid tumors	Avelumab	PD-L1	Talazoparib	ORR: 18.2% (95% CI: 5.2%-40.3%) (TNBC); 34.8% (95% CI: 16.4%-57.3%) (HR-positive, ERBB2-negative, and DDR-positive BC); 63.6% (95% CI: 30.8%-89.1%) (platinum-sensitive, BRCA1/2-altered OC)	NCT03330405	1/2	[184]

Abbreviation: EOC, epithelial ovarian cancer; PPC, primary peritoneal cancer; OC, ovarian cancer; BC, breast cancer; PDAC, pancreatic ductal adenocarcinoma; CRC, colorectal cancer; SCLC, small cell lung cancer; NSCLC, non-small cell lung cancer; GEJ, esophagogastric junction; TNBC, triple-negative breast cancer; ORR, overall response rate; DCR, disease control rate; PFS, progression free survival; mPFS, median progression free survival; OS, overall survival; mOS, median overall survival

The MEDIOLA trial is a phase 1/2, multi-center, basket trial that evaluates durvalumab and olaparib in four cohorts, including gBRCAm metastatic breast cancer, gBRCAm metastatic ovarian cancer, metastatic gastric cancer, and relapsed small-cell lung cancer (NCT02734004). In the breast cancer cohort, 34 patients are enrolled and receive 300 mg BID of olaparib orally for 4 weeks followed by a combination of 300 mg BID of olaparib orally and 1.5 g durvalumab through intravenous infusion once every 4 weeks. All patients are included in the safety analysis, among which 30 patients are eligible for efficacy analysis. The analysis results demonstrate that the safety and tolerability of the combination are acceptable, and the 12-week disease control rate (DCR) reaches 80% [178]. In the ovarian cancer cohort, 32 patients are enrolled and receive the same modality of treatment as the breast cancer cohort. Similarly, the treatment is well tolerated, and the 12-week DCR achieves 90% [179]. Updated data shows an ORR of 71.9%, a median PFS of 11.1 months, a median duration of response (DoR) of 10.2 months, and a DCR at 28 weeks of 65.6% [180]. Furthermore, a total of 51 gBRCAm patients are included in the expansion cohort of ovarian cancer, the ORR of which is 92.2% [181].

In contrast, in the JAVELIN BRCA/ATM, a phase 2b, multi-center trial that evaluates avelumab plus talazoparib in patients with advanced BRCA1/2-mutated or ATM-mutated solid tumors (NCT03565991), the ORR of the BRCA1/2 cohort (159 patients) and the ATM cohort (41 patients) are only 26.4% and 4.9%, respectively [182].

Besides, in the phase 2 TOPACIO trial that assesses the combination of niraparib and pembrolizumab in patients with advanced or metastatic TNBC (NCT02657889), the BRCA-mutated cohort (15 patients) exhibits an ORR of 47%, a DCR of 80%, and a median PFS of 8.3 months, while the BRCA-WT cohort (27 patients) exhibits an ORR of 11%, a DCR of 33%, and a median PFS of 2.1 months [183]. This result is consistent with the finding of the JAVELIN PARP Medley trial that patients with BRCA alterations respond more sensitively to combined ICIs and PARPi treatment compared with patients with BRCA-WT tumors (NCT03330405) [184].

In spite of the evidence from the non-randomized MEDIOLA, JAVELIN, and TOPACIO trials indicating the encouraging safety and activity of ICIs plus PARPi in HRD cancers, further assessment in randomized trials to specify whether the combined use of ICIs could improve the efficacy of PARPi is warranted. Indeed, in a phase 2 randomized trial of BRCA1/2 mutated, locally advanced or metastatic non-HER2-positive breast cancer (NCT02849496), 78 patients are divided into two cohorts, one treated with Olaparib, and the other treated with Olaparib plus atezolizumab. Similar efficacy is observed in the two cohorts (PFS: 7.0 vs. 7.67 months; median OS: 26.5 vs. 22.4 months) [185].

Taken together, the benefits of ICIs for HRD cancers are not conclusively defined, constrained by the scale and design of current clinical trials. More definitive evidence requires further validation in larger clinical trials.

## Conclusions

In this review, focusing on four types of HRD cancers, we summarize the intricate interactions and crosstalk between immune cells and tumor cells, as well as among various signaling pathways within the TME, depicting a distinct immune microenvironment landscape in HRD cancers. A comprehensive understanding of the characteristics of HRD cancers could provide theoretical support and evidence for precisely targeting immunosuppressive factors within the TME. Additionally, we highlight recent advances in the combination of PARPi and immunotherapies across various preclinical models and clinical trials of HRD cancers, which represents a feasible combined therapeutic strategy for the treatment of HRD cancers.

**Fig. 4:**
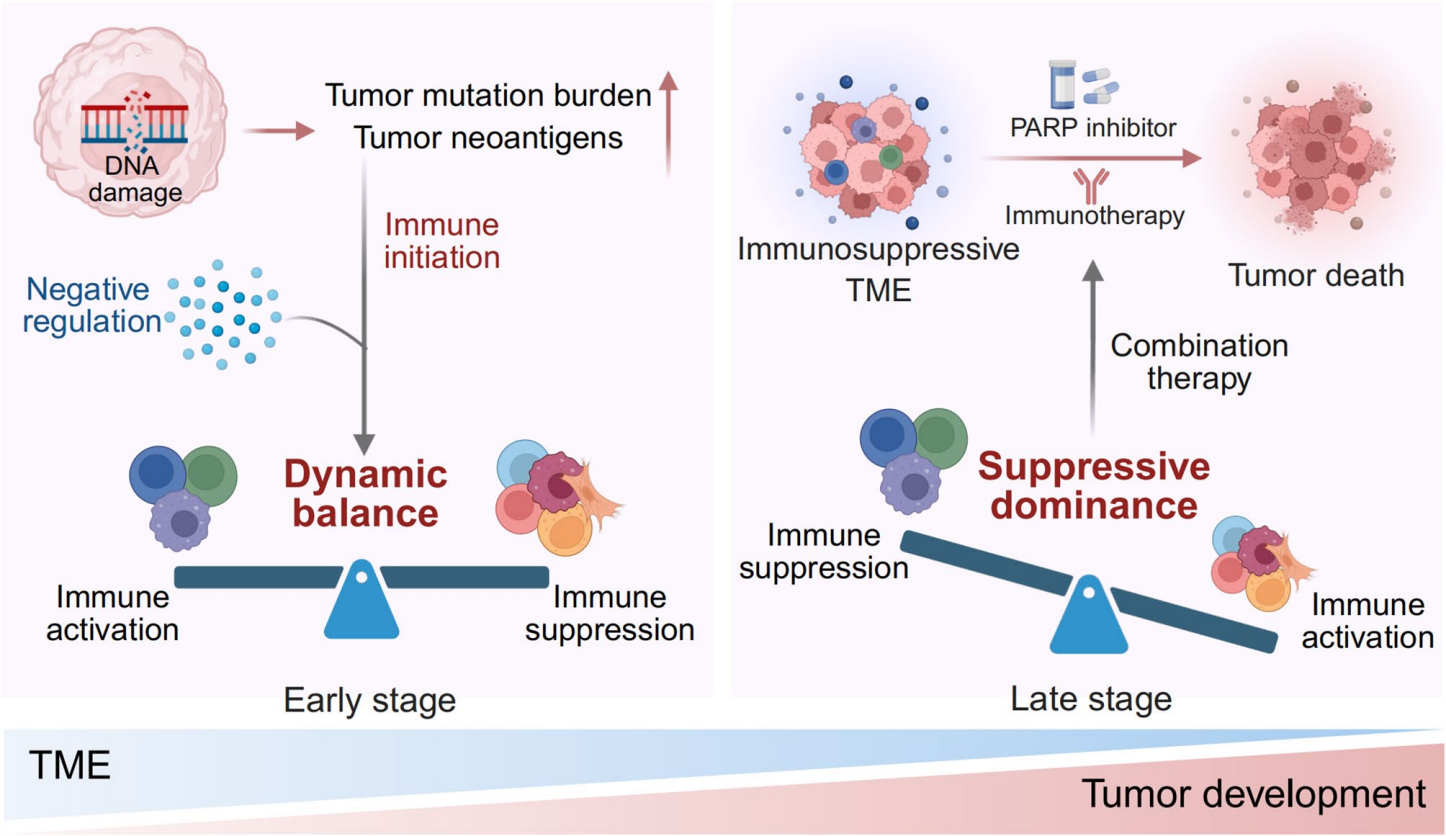
Clinical significance of the distinct TME of HRD cancers. Immune activation and immune suppression co-exist in a dynamic balance during the development of HRD cancers. At the late stage, however, negative immune regulation predominates, resulting in the formation of immunosuppressive microenvironment. The combined pharmacotherapy of PARP inhibitors and immunotherapies holds significant promise to bring novel breakthroughs in the treatment of HRD cancers. HRD, homologous recombination deficiency; TME, tumor microenvironment; PARP, poly-(ADP-ribose) polymerase

The unique TME of HRD cancers is characterized by the coexistence of immune activation and immune suppression, with a dynamic balance between these two forces that evolves during the initiation and progression of HRD cancers. Starting from individuals harboring HRR-related gene defects but not yet developing tumors, the microenvironment at the site of tumor initiation already exhibits lymphocyte enrichment, indicating the establishment of early anti-tumor immunity. The increased TMB and neoantigen load in HRD cancers lead to active antigen presentation within the immune microenvironment. This results in a more substantial infiltration of diverse immune cells, including CD4^+^/CD8^+^ T cells, NK cells, B cells, and macrophages. Collectively, these observations suggest that the TME of HRD cancers displays a robustly immune-activated status in the early stages. Nonetheless, evidence indicates that the TME of HRD cancers concurrently harbors negative regulatory immune elements. In the early microenvironment of the fallopian tubes or breasts in patients with HRR gene defects, there is an upregulation of co-inhibitory molecules such as PD-1, LAG-3, and TIGIT, along with an increase in exhausted lymphocytes. This suggests that while adaptive immune response is activated at the early stage, immune exhaustion also occurs simultaneously. This phenomenon may elucidate the more rapid progression observed in breast and ovarian cancers among patients with HRR gene defects. Furthermore, within the TME of HRD cancers, negative immune regulation is also noted, characterized by increased numbers of Treg cells, exhausted lymphocytes, and myeloid and stromal cells expressing co-inhibitory molecules. The immune activation and suppression within the TME co-exist in a state of dynamic equilibrium during the development of HRD cancers. At the late stage of HRD cancers, however, negative immune regulation assumes a preponderant role, resulting in the formation of an immunosuppressive microenvironment. This provides solid evidence supporting the combination of PARPi and immunotherapies, especially ICIs, for treating HRD cancers (Fig. 4).

Substantial progress has been attained in the application of immunotherapies in HRD cancers. This includes not only a deeper understanding of the underlying molecular mechanisms that explain the interplay among immune checkpoints, HRR pathway, and PARPi, but also the successful translation of preclinical findings into promising clinical trials, thereby paving the way for more effective treatment strategies in HRD cancers. In addition to ICIs, strategies aimed at targeting other immunoregulatory elements (including Treg cells, macrophages, CAFs, and cGAS-STING signaling) within the TME, are currently undergoing progressive and in-depth exploration, and demonstrate favorable synergistic efficacy with PARPi in preclinical models. The combined pharmacotherapy of PARPi and immunotherapies holds significant promise to bring novel breakthroughs in the treatment of HRD cancers.

## Data Availability

No datasets were generated or analysed during the current study.

## Abbreviations

HRD: Homologous recombination deficiency
HRR: Homologous recombination repair
PARP: Poly-(ADP-ribose) polymerase
PARPi: Poly-(ADP-ribose) polymerase inhibitor
TME: Tumor microenvironment
DSB: Double-strand break
NHEJ: Non-homologous end joining
MMEJ: Microhomology-mediated end joining
SSA: Single-strand annealing
TMB: Tumor mutation burden
APC: Antigen-presenting cell
Treg: Regulatory T cell
PFS: Progression-free survival
OS: Overall survival
gBRCA: Germline BRCA1 and BRCA2
sBRCA: Somatic BRCA1 and BRCA2
LST: Large-scale transitions
LOH: Loss of heterozygosity
TAI: Telomeric allelic imbalance
GIS: Genomic instability score
HGSOC: High grade serous ovarian cancer
TNBC: Triple-negative breast cancer
PDAC: Pancreatic ductal adenocarcinoma
PACC: Pancreatic acinar cell carcinoma
NSCLC: Non-small-cell lung cancer
HRP: Homologous recombination proficient
TIL: Tumor infiltrating lymphocyte
Trm: Tissue resident memory T cell
TIS: T cell inflammation signature
Tcm: Central memory T cell
Tem: Effector memory T cell
ICB: Immune checkpoint blockade
WT: Wild type
eTreg: Effector Treg
Tex: Exhausted T cell
CIS: Clustered immune space
FIS: Free immune space
TIL-B: Tumor-infiltrating B lymphocyte
NK: Natural killer
TAM: Tumor associated macrophage
DC: Dendritic cell
CAF: Cancer-associated fibroblast
ECM: Extracellular matrix
MMP: Matrix metalloproteinase
apCAF: Antigen presentation associated fibroblast
myCAF: Myofibroblastic CAF
IFN: Interferon
OXPHOS: Oxidative phosphorylation
PFI: Platinum-free Interval
MPR: Major pathologic response
ACT: Anthracycline, cyclophosphamide and taxane
FFI: Failure-free interval
DSS: Disease-specific survival
ORR: Overall response rate
mAb: Monoclonal antibody
EMT: Epithelial-mesenchymal transition
SIRP-: Ligand signal regulatory protein
SASP: Senescence-associated secretory phenotype
ADCC: Antibody-dependent cellular cytotoxicity
ICI: Immune checkpoint inhibitor

## References

[1] Mouw KW, Goldberg MS, Konstantinopoulos PA, D’Andrea AD. DNA damage and repair biomarkers of immunotherapy response. Cancer Discov. 2017;7:675–93. 28630051 10.1158/2159-8290.CD-17-0226 PMC5659200

[2] Knijnenburg TA, Wang L, Zimmermann MT, Chambwe N, Gao GF, Cherniack AD, et al. Genomic and molecular landscape of DNA damage repair deficiency across the cancer genome atlas. Cell Rep. 2018;23:239–e2546. 29617664 10.1016/j.celrep.2018.03.076 PMC5961503

[3] Lei T, Du S, Peng Z, Chen L. Multifaceted regulation and functions of 53BP1 in NHEJ–mediated DSB repair (Review). Int J Mol Med. 2022;50:90. 35583003 10.3892/ijmm.2022.5145 PMC9162042

[4] Kadam A, Shilo S, Naor H, Wainstein A, Brilon Y, Feldman T, et al. Utilizing insights of DNA repair machinery to discover MMEJ deletions and novel mechanisms. Nucleic Acids Res. 2024;52:e106. 39607705 10.1093/nar/gkae1132 PMC11662932

[5] Setton J, Hadi K, Choo Z-N, Kuchin KS, Tian H, Da Cruz Paula A, et al. Long-molecule scars of backup DNA repair in BRCA1- and BRCA2-deficient cancers. Nature. 2023;621:129–37. 37587346 10.1038/s41586-023-06461-2 PMC10482687

[6] Her J, Bunting SF. How cells ensure correct repair of DNA double-strand breaks. J Biol Chem. 2018;293:10502–11. 29414795 10.1074/jbc.TM118.000371 PMC6036189

[7] Sfeir A, Symington LS. Microhomology-mediated end joining: a back-up survival mechanism or dedicated pathway? Trends Biochem Sci. 2015;40:701–14. 26439531 10.1016/j.tibs.2015.08.006 PMC4638128

[8] Kechin A, Koryukov M, Mikheeva R, Filipenko M. Homologous recombination deficiency (HRD) diagnostics: underlying mechanisms and new perspectives. Cancer Metastasis Rev. 2024;44:19. 39724448 10.1007/s10555-024-10238-y

[9] Huang Y, Qiu Y, Ding L, Ren S, Jiang Y, Luo J, et al. Somatic mutations in four novel genes contribute to homologous recombination deficiency in breast cancer: a real-world clinical tumor sequencing study. J Pathol Clin Res. 2024;10:e12367. 38504382 10.1002/2056-4538.12367 PMC10951049

[10] Maxwell KN, Domchek SM, Nathanson KL, Robson ME. Population frequency of germline BRCA1/2 mutations. J Clin Oncol. 2016;34:4183–5. 27551127 10.1200/JCO.2016.67.0554

[11] Lord CJ, Ashworth A. BRCAness revisited. Nat Rev Cancer. 2016;16:110–20. 26775620 10.1038/nrc.2015.21

[12] McCabe N, Turner NC, Lord CJ, Kluzek K, Bialkowska A, Swift S, et al. Deficiency in the repair of DNA damage by homologous recombination and sensitivity to poly(ADP-ribose) polymerase Inhibition. Cancer Res. 2006;66:8109–15. 16912188 10.1158/0008-5472.CAN-06-0140

[13] Murai J, Pommier Y, BRCAness. Homologous recombination deficiencies, and synthetic lethality. Cancer Res. 2023;83:1173–4. 37057596 10.1158/0008-5472.CAN-23-0628

[14] Yoshida R, Hagio T, Kaneyasu T, Gotoh O, Osako T, Tanaka N, et al. Pathogenicity assessment of variants for breast cancer susceptibility genes based on BRCAness of tumor sample. Cancer Sci. 2021;112:1310–9. 33421217 10.1111/cas.14803 PMC7935793

[15] Nielsen FC, van Overeem Hansen T, Sørensen CS. Hereditary breast and ovarian cancer: new genes in confined pathways. Nat Rev Cancer. 2016;16:599–612. 27515922 10.1038/nrc.2016.72

[16] Dhawan M, Ryan CJ, Ashworth A. DNA repair deficiency is common in advanced prostate cancer: new therapeutic opportunities. Oncologist. 2016;21:940–5. 27317574 10.1634/theoncologist.2016-0135 PMC4978560

[17] Hu Y, Guo M. Synthetic lethality strategies: beyond BRCA1/2 mutations in pancreatic cancer. Cancer Sci. 2020;111:3111–21. 32639661 10.1111/cas.14565 PMC7469842

[18] Robinson D, Van Allen EM, Wu Y-M, Schultz N, Lonigro RJ, Mosquera J-M, et al. Integrative clinical genomics of advanced prostate cancer. Cell. 2015;161:1215–28. 26000489 10.1016/j.cell.2015.05.001 PMC4484602

[19] Roberts NJ, Jiao Y, Yu J, Kopelovich L, Petersen GM, Bondy ML, et al. ATM mutations in patients with hereditary pancreatic cancer. Cancer Discov. 2012;2:41–6. 22585167 10.1158/2159-8290.CD-11-0194 PMC3676748

[20] Waddell N, Pajic M, Patch A-M, Chang DK, Kassahn KS, Bailey P, et al. Whole genomes redefine the mutational landscape of pancreatic cancer. Nature. 2015;518:495–501. 25719666 10.1038/nature14169 PMC4523082

[21] Farmer H, McCabe N, Lord CJ, Tutt ANJ, Johnson DA, Richardson TB, et al. Targeting the DNA repair defect in BRCA mutant cells as a therapeutic strategy. Nature. 2005;434:917–21. 15829967 10.1038/nature03445

[22] Bryant HE, Schultz N, Thomas HD, Parker KM, Flower D, Lopez E, et al. Specific killing of BRCA2-deficient tumours with inhibitors of poly(ADP-ribose) polymerase. Nature. 2005;434:913–7. 15829966 10.1038/nature03443

[23] Pokataev I, Fedyanin M, Polyanskaya E, Popova A, Agafonova J, Menshikova S, et al. Efficacy of platinum-based chemotherapy and prognosis of patients with pancreatic cancer with homologous recombination deficiency: comparative analysis of published clinical studies. ESMO Open. 2020;5:e000578. 33551067 10.1136/esmoopen-2019-000578 PMC7003386

[24] Patel PS, Algouneh A, Hakem R. Exploiting synthetic lethality to target BRCA1/2-deficient tumors: where we stand. Oncogene. 2021;40:3001–14. 33716297 10.1038/s41388-021-01744-2

[25] Mateo J, Lord CJ, Serra V, Tutt A, Balmaña J, Castroviejo-Bermejo M, et al. A decade of clinical development of PARP inhibitors in perspective. Ann Oncol. 2019;30:1437–47. 31218365 10.1093/annonc/mdz192 PMC6771225

[26] Chou J, Robinson TM, Egusa EA, Lodha R, Zhang M, Badura M, et al. Synthetic lethal targeting of CDK12-deficient prostate cancer with PARP inhibitors. Clin Cancer Res. 2024;30:5445–58. 39321214 10.1158/1078-0432.CCR-23-3785 PMC11611633

[27] LaFargue CJ, Dal Molin GZ, Sood AK, Coleman RL. Exploring and comparing adverse events between PARP inhibitors. Lancet Oncol. 2019;20:e15–28. 30614472 10.1016/S1470-2045(18)30786-1 PMC7292736

[28] Lord CJ, Ashworth A. PARP inhibitors: synthetic lethality in the clinic. Science. 2017;355:1152–8. 28302823 10.1126/science.aam7344 PMC6175050

[29] Zhou A, Butt O, Ansstas M, Mauer E, Khaddour K, Ansstas G. Determining PARP Inhibition as a treatment strategy in melanoma based on homologous recombination deficiency-related loss of heterozygosity. J Natl Compr Canc Netw. 2023;21:688–e6933. 37433433 10.6004/jnccn.2022.7102

[30] Akinjiyan FA, Morecroft R, Phillipps J, Adeyelu T, Elliott A, Park SJ, et al. Homologous recombination deficiency (HRD) in cutaneous oncology. Int J Mol Sci. 2023;24:10771. 37445949 10.3390/ijms241310771 PMC10341889

[31] Jamieson A, Sobral de Barros J, Cochrane DR, Douglas JM, Shankar S, Lynch BJ, et al. Targeted and shallow whole-genome sequencing identifies therapeutic opportunities in p53abn endometrial cancers. Clin Cancer Res. 2024;30:2461–74. 38536067 10.1158/1078-0432.CCR-23-3689 PMC11145180

[32] Beinse G, Just P-A, Le Frere Belda M-A, Laurent-Puig P, Jacques S, Koual M, et al. Discovery and validation of a transcriptional signature identifying homologous recombination-deficient breast, endometrial and ovarian cancers. Br J Cancer. 2022;127:1123–32. 35752712 10.1038/s41416-022-01900-9 PMC9470569

[33] Serra-Camprubí Q, Verdaguer H, Oliveros W, Lupión-Garcia N, Llop-Guevara A, Molina C, et al. Human metastatic cholangiocarcinoma patient-derived xenografts and tumoroids for preclinical drug evaluation. Clin Cancer Res. 2023;29:432–45. 36374558 10.1158/1078-0432.CCR-22-2551 PMC9873249

[34] Cecchini M, Pilat MJ, Uboha N, Azad NS, Cho M, Davis EJ, et al. Olaparib in treatment-refractory isocitrate dehydrogenase 1 (IDH1)- and IDH2-mutant cholangiocarcinoma: safety and antitumor activity from the phase 2 National cancer Institute 10129 trial. Cancer. 2025;131:e35755. 39917990 10.1002/cncr.35755 PMC11949439

[35] Ichikawa H, Aizawa M, Kano Y, Hanyu T, Muneoka Y, Hiroi S, et al. Landscape of homologous recombination deficiency in gastric cancer and clinical implications for first-line chemotherapy. Gastric Cancer. 2024;27:1273–86. 39110344 10.1007/s10120-024-01542-1

[36] Bzura A, Spicer JB, Dulloo S, Yap TA, Fennell DA. Targeting DNA damage response deficiency in thoracic cancers. Drugs. 2024;84:1025–33. 39001941 10.1007/s40265-024-02066-9

[37] Wang Y, Ma Y, He L, Du J, Li X, Jiao P, et al. Clinical and molecular significance of homologous recombination deficiency positive non-small cell lung cancer in Chinese population: an integrated genomic and transcriptional analysis. Chin J Cancer Res. 2024;36:282–97. 38988485 10.21147/j.issn.1000-9604.2024.03.05 PMC11230889

[38] Launonen I-M, Lyytikäinen N, Casado J, Anttila EA, Szabó A, Haltia U-M, et al. Single-cell tumor-immune microenvironment of BRCA1/2 mutated high-grade serous ovarian cancer. Nat Commun. 2022;13:835. 35149709 10.1038/s41467-022-28389-3 PMC8837628

[39] Nguyen L, Martens WM, Van Hoeck J, Cuppen A. Pan-cancer landscape of homologous recombination deficiency. Nat Commun. 2020;11:5584. 33149131 10.1038/s41467-020-19406-4 PMC7643118

[40] Budczies J, Kluck K, Beck S, Ourailidis I, Allgäuer M, Menzel M, et al. Homologous recombination deficiency is inversely correlated with microsatellite instability and identifies immunologically cold tumors in most cancer types. J Pathol Clin Res. 2022;8:371–82. 35384413 10.1002/cjp2.271 PMC9161338

[41] Chi H, Pepper M, Thomas PG. Principles and therapeutic applications of adaptive immunity. Cell. 2024;187:2052–78. 38670065 10.1016/j.cell.2024.03.037 PMC11177542

[42] Wen WX, Leong C-O. Association of BRCA1- and BRCA2-deficiency with mutation burden, expression of PD-L1/PD-1, immune infiltrates, and T cell-inflamed signature in breast cancer. PLoS ONE. 2019;14:e0215381. 31022191 10.1371/journal.pone.0215381 PMC6483182

[43] Nolan E, Savas P, Policheni AN, Darcy PK, Vaillant F, Mintoff CP, et al. Combined immune checkpoint Blockade as a therapeutic strategy for BRCA1-mutated breast cancer. Sci Transl Med. 2017;9:eaal4922. 28592566 10.1126/scitranslmed.aal4922 PMC5822709

[44] Mandelker D, Marra A, Zheng-Lin B, Selenica P, Blanco-Heredia J, Zhu Y, et al. Genomic profiling reveals germline predisposition and homologous recombination deficiency in pancreatic acinar cell carcinoma. J Clin Oncol. 2023;41:5151–62. 37607324 10.1200/JCO.23.00561 PMC10667000

[45] Vázquez-García I, Uhlitz F, Ceglia N, Lim JLP, Wu M, Mohibullah N, et al. Ovarian cancer mutational processes drive site-specific immune evasion. Nature. 2022;612:778–86. 36517593 10.1038/s41586-022-05496-1 PMC9771812

[46] Paulet L, Trecourt A, Leary A, Peron J, Descotes F, Devouassoux-Shisheboran M, et al. Cracking the homologous recombination deficiency code: how to identify responders to PARP inhibitors. Eur J Cancer. 2022;166:87–99. 35279473 10.1016/j.ejca.2022.01.037

[47] Roy R, Chun J, Powell SN. BRCA1 and BRCA2: different roles in a common pathway of genome protection. Nat Rev Cancer. 2011;12:68–78. 22193408 10.1038/nrc3181 PMC4972490

[48] Kanchi KL, Johnson KJ, Lu C, McLellan MD, Leiserson MDM, Wendl MC, et al. Integrated analysis of germline and somatic variants in ovarian cancer. Nat Commun. 2014;5:3156. 24448499 10.1038/ncomms4156 PMC4025965

[49] Cancer Genome Atlas Research Network. Integrated genomic analyses of ovarian carcinoma. Nature. 2011;474:609–15. 21720365 10.1038/nature10166 PMC3163504

[50] Mirza MR, Monk BJ, Herrstedt J, Oza AM, Mahner S, Redondo A, et al. Niraparib maintenance therapy in platinum-sensitive, recurrent ovarian cancer. N Engl J Med. 2016;375:2154–64. 27717299 10.1056/NEJMoa1611310

[51] Swisher EM, Lin KK, Oza AM, Scott CL, Giordano H, Sun J, et al. Rucaparib in relapsed, platinum-sensitive high-grade ovarian carcinoma (ARIEL2 part 1): an international, multicentre, open-label, phase 2 trial. Lancet Oncol. 2017;18:75–87. 27908594 10.1016/S1470-2045(16)30559-9

[52] Bajrami I, Frankum JR, Konde A, Miller RE, Rehman FL, Brough R, et al. Genome-wide profiling of genetic synthetic lethality identifies CDK12 as a novel determinant of PARP1/2 inhibitor sensitivity. Cancer Res. 2014;74:287–97. 24240700 10.1158/0008-5472.CAN-13-2541 PMC4886090

[53] Loveday C, Turnbull C, Ramsay E, Hughes D, Ruark E, Frankum JR, et al. Germline mutations in RAD51D confer susceptibility to ovarian cancer. Nat Genet. 2011;43:879–82. 21822267 10.1038/ng.893 PMC4845885

[54] Kondrashova O, Nguyen M, Shield-Artin K, Tinker AV, Teng NNH, Harrell MI, et al. Secondary somatic mutations restoring RAD51C and RAD51D associated with acquired resistance to the PARP inhibitor Rucaparib in high-grade ovarian carcinoma. Cancer Discov. 2017;7:984–98. 28588062 10.1158/2159-8290.CD-17-0419 PMC5612362

[55] Chang HHY, Pannunzio NR, Adachi N, Lieber MR. Non-homologous DNA end joining and alternative pathways to double-strand break repair. Nat Rev Mol Cell Biol. 2017;18:495–506. 28512351 10.1038/nrm.2017.48 PMC7062608

[56] Abkevich V, Timms KM, Hennessy BT, Potter J, Carey MS, Meyer LA, et al. Patterns of genomic loss of heterozygosity predict homologous recombination repair defects in epithelial ovarian cancer. Br J Cancer. 2012;107:1776–82. 23047548 10.1038/bjc.2012.451 PMC3493866

[57] Birkbak NJ, Wang ZC, Kim J-Y, Eklund AC, Li Q, Tian R, et al. Telomeric allelic imbalance indicates defective DNA repair and sensitivity to DNA-damaging agents. Cancer Discov. 2012;2:366–75. 22576213 10.1158/2159-8290.CD-11-0206 PMC3806629

[58] Timms KM, Abkevich V, Hughes E, Neff C, Reid J, Morris B, et al. Association of BRCA1/2 defects with genomic scores predictive of DNA damage repair deficiency among breast cancer subtypes. Breast Cancer Res. 2014;16:475. 25475740 10.1186/s13058-014-0475-x PMC4308910

[59] Marquard AM, Eklund AC, Joshi T, Krzystanek M, Favero F, Wang ZC, et al. Pan-cancer analysis of genomic Scar signatures associated with homologous recombination deficiency suggests novel indications for existing cancer drugs. Biomark Res. 2015;3:9. 26015868 10.1186/s40364-015-0033-4 PMC4443545

[60] Telli ML, Timms KM, Reid J, Hennessy B, Mills GB, Jensen KC, et al. Homologous recombination deficiency (HRD) score predicts response to platinum-containing neoadjuvant chemotherapy in patients with triple-negative breast cancer. Clin Cancer Res. 2016;22:3764–73. 26957554 10.1158/1078-0432.CCR-15-2477 PMC6773427

[61] Frampton GM, Fichtenholtz A, Otto GA, Wang K, Downing SR, He J, et al. Development and validation of a clinical cancer genomic profiling test based on massively parallel DNA sequencing. Nat Biotechnol. 2013;31:1023–31. 24142049 10.1038/nbt.2696 PMC5710001

[62] Pellegrino B, Herencia-Ropero A, Llop-Guevara A, Pedretti F, Moles-Fernández A, Viaplana C, et al. Preclinical in vivo validation of the RAD51 test for identification of homologous recombination-deficient tumors and patient stratification. Cancer Res. 2022;82:1646–57. 35425960 10.1158/0008-5472.CAN-21-2409 PMC7612637

[63] Castroviejo-Bermejo M, Cruz C, Llop-Guevara A, Gutiérrez-Enríquez S, Ducy M, Ibrahim YH, et al. A RAD51 assay feasible in routine tumor samples calls PARP inhibitor response beyond BRCA mutation. EMBO Mol Med. 2018;10:e9172. 30377213 10.15252/emmm.201809172 PMC6284440

[64] Foo TK, Xia B. BRCA1-dependent and independent recruitment of PALB2-BRCA2-RAD51 in the DNA damage response and cancer. Cancer Res. 2022;82:3191–7. 35819255 10.1158/0008-5472.CAN-22-1535 PMC9481714

[65] Mukhopadhyay A, Plummer ER, Elattar A, Soohoo S, Uzir B, Quinn JE, et al. Clinicopathological features of homologous recombination-deficient epithelial ovarian cancers: sensitivity to PARP inhibitors, platinum, and survival. Cancer Res. 2012;72:5675–82. 23066035 10.1158/0008-5472.CAN-12-0324

[66] Hill SJ, Decker B, Roberts EA, Horowitz NS, Muto MG, Worley MJ, et al. Prediction of DNA repair inhibitor response in short-term patient-derived ovarian cancer organoids. Cancer Discov. 2018;8:1404–21. 30213835 10.1158/2159-8290.CD-18-0474 PMC6365285

[67] Graeser M, McCarthy A, Lord CJ, Savage K, Hills M, Salter J, et al. A marker of homologous recombination predicts pathologic complete response to neoadjuvant chemotherapy in primary breast cancer. Clin Cancer Res. 2010;16:6159–68. 20802015 10.1158/1078-0432.CCR-10-1027 PMC3432445

[68] Meijer TG, Verkaik NS, Sieuwerts AM, van Riet J, Naipal KAT, van Deurzen CHM, et al. Functional ex vivo assay reveals homologous recombination deficiency in breast cancer beyond BRCA gene defects. Clin Cancer Res. 2018;24:6277–87. 30139880 10.1158/1078-0432.CCR-18-0063

[69] Siegel RL, Kratzer TB, Giaquinto AN, Sung H, Jemal A. Cancer statistics, 2025. CA Cancer J Clin. 2025;75:10–45. 39817679 10.3322/caac.21871 PMC11745215

[70] Lheureux S, Gourley C, Vergote I, Oza AM. Epithelial ovarian cancer. Lancet. 2019;393:1240–53. 30910306 10.1016/S0140-6736(18)32552-2

[71] Colombo P-E, Taoum C, Fabbro M, Quesada S, Rouanet P, Ray-Coquard I. Impact of molecular testing on the surgical management of advanced epithelial ovarian cancer. Crit Rev Oncol Hematol. 2024;202:104469. 39111459 10.1016/j.critrevonc.2024.104469

[72] Ngoi NYL, Tan DSP. The role of homologous recombination deficiency testing in ovarian cancer and its clinical implications: do we need it? ESMO Open. 2021;6:100144. 34015643 10.1016/j.esmoop.2021.100144 PMC8141874

[73] Burdett NL, Willis MO, Alsop K, Hunt AL, Pandey A, Hamilton PT, et al. Multiomic analysis of homologous recombination-deficient end-stage high-grade serous ovarian cancer. Nat Genet. 2023;55:437–50. 36849657 10.1038/s41588-023-01320-2

[74] Bray F, Laversanne M, Sung H, Ferlay J, Siegel RL, Soerjomataram I, et al. Global cancer statistics 2022: GLOBOCAN estimates of incidence and mortality worldwide for 36 cancers in 185 countries. CA Cancer J Clin. 2024;74:229–63. 38572751 10.3322/caac.21834

[75] Tufail M. DNA repair pathways in breast cancer: from mechanisms to clinical applications. Breast Cancer Res Treat. 2023;200:305–21. 37289340 10.1007/s10549-023-06995-z

[76] Couch FJ, Hart SN, Sharma P, Toland AE, Wang X, Miron P, et al. Inherited mutations in 17 breast cancer susceptibility genes among a large triple-negative breast cancer cohort unselected for family history of breast cancer. J Clin Oncol. 2015;33:304–11. 25452441 10.1200/JCO.2014.57.1414 PMC4302212

[77] Davies H, Glodzik D, Morganella S, Yates LR, Staaf J, Zou X, et al. HRDetect is a predictor of BRCA1 and BRCA2 deficiency based on mutational signatures. Nat Med. 2017;23:517–25. 28288110 10.1038/nm.4292 PMC5833945

[78] Jacobson DH, Pan S, Fisher J, Secrier M. Multi-scale characterisation of homologous recombination deficiency in breast cancer. Genome Med. 2023;15:90. 37919776 10.1186/s13073-023-01239-7 PMC10621207

[79] Arce-Gallego S, Cresta Morgado P, Delgado-Serrano L, Simonetti S, Gonzalez M, Romero-Lozano P et al. Homologous recombination repair status in metastatic prostate cancer by next-generation sequencing and functional Immunofluorescence. Cell Rep Med. 2025;101937. 10.1016/j.xcrm.2025.101937 PMC11866514 39914385

[80] Lotan TL, Kaur HB, Salles DC, Murali S, Schaeffer EM, Lanchbury JS, et al. Homologous recombination deficiency (HRD) score in germline BRCA2- versus ATM-altered prostate cancer. Mod Pathol. 2021;34:1185–93. 33462368 10.1038/s41379-020-00731-4 PMC8154637

[81] Pritchard CC, Mateo J, Walsh MF, De Sarkar N, Abida W, Beltran H, et al. Inherited DNA-repair gene mutations in men with metastatic prostate cancer. N Engl J Med. 2016;375:443–53. 27433846 10.1056/NEJMoa1603144 PMC4986616

[82] Easton DF, Pharoah PDP, Antoniou AC, Tischkowitz M, Tavtigian SV, Nathanson KL, et al. Gene-panel sequencing and the prediction of breast-cancer risk. N Engl J Med. 2015;372:2243–57. 26014596 10.1056/NEJMsr1501341 PMC4610139

[83] Naro C, Ruta V, Sette C. Splicing dysregulation: hallmark and therapeutic opportunity in pancreatic cancer. Trends Mol Med. 2024. S1471-4914(24)00308-3. 10.1016/j.molmed.2024.11.007 39648052

[84] Kindler HL, Hammel P, Reni M, Van Cutsem E, Macarulla T, Hall MJ, et al. Overall survival results from the POLO trial: a phase III study of active maintenance Olaparib versus placebo for germline BRCA-mutated metastatic pancreatic cancer. J Clin Oncol. 2022;40:3929–39. 35834777 10.1200/JCO.21.01604 PMC10476841

[85] Casolino R, Paiella S, Azzolina D, Beer PA, Corbo V, Lorenzoni G, et al. Homologous recombination deficiency in pancreatic cancer: a systematic review and prevalence meta-analysis. J Clin Oncol. 2021;39:2617–31. 34197182 10.1200/JCO.20.03238 PMC8331063

[86] Rempel E, Kluck K, Beck S, Ourailidis I, Kazdal D, Neumann O, et al. Pan-cancer analysis of genomic Scar patterns caused by homologous repair deficiency (HRD). NPJ Precis Oncol. 2022;6:36. 35681079 10.1038/s41698-022-00276-6 PMC9184602

[87] Konstantinopoulos PA, Ceccaldi R, Shapiro GI, D’Andrea AD. Homologous recombination deficiency: exploiting the fundamental vulnerability of ovarian cancer. Cancer Discov. 2015;5:1137–54. 26463832 10.1158/2159-8290.CD-15-0714 PMC4631624

[88] Bluestone JA, Abbas AK. Natural versus adaptive regulatory T cells. Nat Rev Immunol. 2003;3:253–7. 12658273 10.1038/nri1032

[89] Oliveira G, Wu CJ. Dynamics and specificities of T cells in cancer immunotherapy. Nat Rev Cancer. 2023;23:295–316. 37046001 10.1038/s41568-023-00560-y PMC10773171

[90] Jin M-Z, Jin W-L. The updated landscape of tumor microenvironment and drug repurposing. Signal Transduct Target Ther. 2020;5:166. 32843638 10.1038/s41392-020-00280-x PMC7447642

[91] Speiser DE, Ho P-C, Verdeil G. Regulatory circuits of T cell function in cancer. Nat Rev Immunol. 2016;16:599–611. 27526640 10.1038/nri.2016.80

[92] Li G, Li S, Jiang Y, Chen T, An Z. Unleashing the power of immune checkpoints: A new strategy for enhancing Treg cells depletion to boost antitumor immunity. Int Immunopharmacol. 2025;147:113952. 39764997 10.1016/j.intimp.2024.113952

[93] Bejarano L, Jordāo MJC, Joyce JA. Therapeutic targeting of the tumor microenvironment. Cancer Discov. 2021;11:933–59. 33811125 10.1158/2159-8290.CD-20-1808

[94] Pagès F, Galon J, Dieu-Nosjean M-C, Tartour E, Sautès-Fridman C, Fridman W-H. Immune infiltration in human tumors: a prognostic factor that should not be ignored. Oncogene. 2010;29:1093–102. 19946335 10.1038/onc.2009.416

[95] Yu X, Lin W, Spirtos A, Wang Y, Chen H, Ye J, et al. Dissection of transcriptome dysregulation and immune characterization in women with germline BRCA1 mutation at single-cell resolution. BMC Med. 2022;20:283. 36076202 10.1186/s12916-022-02489-9 PMC9461201

[96] Reed AD, Pensa S, Steif A, Stenning J, Kunz DJ, Porter LJ, et al. A single-cell atlas enables mapping of homeostatic cellular shifts in the adult human breast. Nat Genet. 2024;56:652–62. 38548988 10.1038/s41588-024-01688-9 PMC11018528

[97] Garsed DW, Pandey A, Fereday S, Kennedy CJ, Takahashi K, Alsop K, et al. The genomic and immune landscape of long-term survivors of high-grade serous ovarian cancer. Nat Genet. 2022;54:1853–64. 36456881 10.1038/s41588-022-01230-9 PMC10478425

[98] Kaur HB, Vidotto T, Mendes AA, Salles DC, Isaacs WB, Antonarakis ES, et al. Association between pathogenic germline mutations in BRCA2 and ATM and tumor-infiltrating lymphocytes in primary prostate cancer. Cancer Immunol Immunother. 2022;71:943–51. 34533610 10.1007/s00262-021-03050-y PMC9254167

[99] Trigos AS, Pasam A, Banks P, Wallace R, Guo C, Keam S, et al. Tumor immune microenvironment of primary prostate cancer with and without germline mutations in homologous recombination repair genes. J Immunother Cancer. 2022;10:e003744. 35764368 10.1136/jitc-2021-003744 PMC9240881

[100] Weeden CE, Gayevskiy V, Marceaux C, Batey D, Tan T, Yokote K, et al. Early immune pressure initiated by tissue-resident memory T cells sculpts tumor evolution in non-small cell lung cancer. Cancer Cell. 2023;41:837–e8526. 37086716 10.1016/j.ccell.2023.03.019

[101] Qiu J, Ren T, Liu Q, Jiang Q, Wu T, Cheng LC, et al. Dissecting the distinct tumor microenvironments of HRD and HRP ovarian cancer: implications for targeted therapies to overcome PARPi resistance in HRD tumors and refractoriness in HRP tumors. Adv Sci (Weinh). 2024;11:e2309755. 39136172 10.1002/advs.202309755 PMC11481286

[102] Perry AS, Annis JS, Master H, Nayor M, Hughes A, Kouame A, et al. Association of longitudinal activity measures and diabetes risk: an analysis from the National institutes of health all of Us research program. J Clin Endocrinol Metab. 2023;108:1101–9. 36458881 10.1210/clinem/dgac695 PMC10306083

[103] Jenzer M, Keß P, Nientiedt C, Endris V, Kippenberger M, Leichsenring J, et al. The BRCA2 mutation status shapes the immune phenotype of prostate cancer. Cancer Immunol Immunother. 2019;68:1621–33. 31549213 10.1007/s00262-019-02393-x PMC6805809

[104] Mehta AK, Cheney EM, Hartl CA, Pantelidou C, Oliwa M, Castrillon JA, et al. Targeting immunosuppressive macrophages overcomes PARP inhibitor resistance in BRCA1-associated triple-negative breast cancer. Nat Cancer. 2021;2:66–82. 33738458 10.1038/s43018-020-00148-7 PMC7963404

[105] Luo Y, Xia Y, Liu D, Li X, Li H, Liu J, et al. Neoadjuvant PARPi or chemotherapy in ovarian cancer informs targeting effector Treg cells for homologous-recombination-deficient tumors. Cell. 2024;187:4905–e492524. 38971151 10.1016/j.cell.2024.06.013

[106] Parkes EE, Walker SM, Taggart LE, McCabe N, Knight LA, Wilkinson R, et al. Activation of STING-dependent innate immune signaling by S-phase-specific DNA damage in breast cancer. J Natl Cancer Inst. 2017;109:djw199. 27707838 10.1093/jnci/djw199 PMC5441301

[107] Shi X, Cheng X, Jiang A, Shi W, Zhu L, Mou W, et al. Immune checkpoints in B cells: unlocking new potentials in cancer treatment. Adv Sci (Weinh). 2024;11:e2403423. 39509319 10.1002/advs.202403423 PMC11653663

[108] Xue D, Hu S, Zheng R, Luo H, Ren X. Tumor-infiltrating B cells: their dual mechanistic roles in the tumor microenvironment. Biomed Pharmacother. 2024;179:117436. 39270540 10.1016/j.biopha.2024.117436

[109] Flippot R, Teixeira M, Rey-Cardenas M, Carril-Ajuria L, Rainho L, Naoun N, et al. B cells and the coordination of immune checkpoint inhibitor response in patients with solid tumors. J Immunother Cancer. 2024;12:e008636. 38631710 10.1136/jitc-2023-008636 PMC11029261

[110] Hui L, Li Y, Huang M-K, Jiang Y-M, Liu T. CXCL13: a common target for immune-mediated inflammatory diseases. Clin Exp Med. 2024;24:244. 39443356 10.1007/s10238-024-01508-8 PMC11499446

[111] Workel HH, Lubbers JM, Arnold R, Prins TM, van der Vlies P, de Lange K, et al. A transcriptionally distinct CXCL13 + CD103 + CD8 + T-cell population is associated with B-cell recruitment and neoantigen load in human cancer. Cancer Immunol Res. 2019;7:784–96. 30872264 10.1158/2326-6066.CIR-18-0517

[112] Wang B, Wang M, Ao D, Wei X. CXCL13-CXCR5 axis: regulation in inflammatory diseases and cancer. Biochim Biophys Acta Rev Cancer. 2022;1877:188799. 36103908 10.1016/j.bbcan.2022.188799

[113] Sautès-Fridman C, Petitprez F, Calderaro J, Fridman WH. Tertiary lymphoid structures in the era of cancer immunotherapy. Nat Rev Cancer. 2019;19:307–25. 31092904 10.1038/s41568-019-0144-6

[114] Laumont CM, Banville AC, Gilardi M, Hollern DP, Nelson BH. Tumour-infiltrating B cells: immunological mechanisms, clinical impact and therapeutic opportunities. Nat Rev Cancer. 2022;22:414–30. 35393541 10.1038/s41568-022-00466-1 PMC9678336

[115] Hong W-F, Zhang F, Wang N, Bi J-M, Zhang D-W, Wei L-S, et al. Dynamic immunoediting by macrophages in homologous recombination deficiency-stratified pancreatic ductal adenocarcinoma. Drug Resist Updat. 2024;76:101115. 39002266 10.1016/j.drup.2024.101115

[116] Myers JA, Miller JS. Exploring the NK cell platform for cancer immunotherapy. Nat Rev Clin Oncol. 2021;18:85–100. 32934330 10.1038/s41571-020-0426-7 PMC8316981

[117] Tarannum M, Romee R, Shapiro RM. Innovative strategies to improve the clinical application of NK cell-based immunotherapy. Front Immunol. 2022;13:859177. 35401529 10.3389/fimmu.2022.859177 PMC8990319

[118] Cursons J, Souza-Fonseca-Guimaraes F, Foroutan M, Anderson A, Hollande F, Hediyeh-Zadeh S, et al. A gene signature predicting natural killer cell infiltration and improved survival in melanoma patients. Cancer Immunol Res. 2019;7:1162–74. 31088844 10.1158/2326-6066.CIR-18-0500

[119] Cappello S, Sung H-M, Ickes C, Gibhardt CS, Vultur A, Bhat H, et al. Protein signatures of NK cell-mediated melanoma killing predict response to immunotherapies. Cancer Res. 2021;81:5540–54. 34518212 10.1158/0008-5472.CAN-21-0164 PMC8727679

[120] Muntasell A, Rojo F, Servitja S, Rubio-Perez C, Cabo M, Tamborero D, et al. NK cell infiltrates and HLA class I expression in primary HER2 + breast cancer predict and uncouple pathological response and disease-free survival. Clin Cancer Res. 2019;25:1535–45. 30523021 10.1158/1078-0432.CCR-18-2365

[121] Huntington ND, Cursons J, Rautela J. The cancer-natural killer cell immunity cycle. Nat Rev Cancer. 2020;20:437–54. 32581320 10.1038/s41568-020-0272-z

[122] Christofides A, Strauss L, Yeo A, Cao C, Charest A, Boussiotis VA. The complex role of tumor-infiltrating macrophages. Nat Immunol. 2022;23:1148–56. 35879449 10.1038/s41590-022-01267-2 PMC10754321

[123] Mantovani A, Marchesi F, Jaillon S, Garlanda C, Allavena P. Tumor-associated myeloid cells: diversity and therapeutic targeting. Cell Mol Immunol. 2021;18:566–78. 33473192 10.1038/s41423-020-00613-4 PMC8027665

[124] Jin R, Neufeld L, McGaha TL. Linking macrophage metabolism to function in the tumor microenvironment. Nat Cancer. 2025. 10.1038/s43018-025-00909-2 39962208

[125] Qorraj M, Bruns H, Böttcher M, Weigand L, Saul D, Mackensen A, et al. The PD-1/PD-L1 axis contributes to immune metabolic dysfunctions of monocytes in chronic lymphocytic leukemia. Leukemia. 2017;31:470–8. 27479178 10.1038/leu.2016.214

[126] Dixon KO, Tabaka M, Schramm MA, Xiao S, Tang R, Dionne D, et al. TIM-3 restrains anti-tumour immunity by regulating inflammasome activation. Nature. 2021;595:101–6. 34108686 10.1038/s41586-021-03626-9 PMC8627694

[127] Seo WI, Lee CH, Jung SJ, Lee DS, Park HY, Jeong DH, et al. Expression of VISTA on tumor-infiltrating immune cells correlated with short intravesical recurrence in non-muscle-invasive bladder cancer. Cancer Immunol Immunother. 2021;70:3113–22. 33770210 10.1007/s00262-021-02906-7 PMC10992450

[128] Jumaniyazova E, Lokhonina A, Dzhalilova D, Miroshnichenko E, Kosyreva A, Fatkhudinov T. The role of macrophages in various types of tumors and the possibility of their use as targets for antitumor therapy. Cancers (Basel). 2025;17:342. 39941714 10.3390/cancers17030342 PMC11815841

[129] Pittet MJ, Michielin O, Migliorini D. Clinical relevance of tumour-associated macrophages. Nat Rev Clin Oncol. 2022;19:402–21. 35354979 10.1038/s41571-022-00620-6

[130] Liu Y, Tan H, Dai J, Lin J, Zhao K, Hu H, et al. Targeting macrophages in cancer immunotherapy: frontiers and challenges. J Adv Res. 2025;S2090–1232(24):00622–2.10.1016/j.jare.2024.12.04339778768

[131] Singer M, Zhang Z, Dayyani F, Zhang Z, Yaghmai V, Choi A, et al. Modulation of tumor-associated macrophages to overcome immune suppression in the hepatocellular carcinoma microenvironment. Cancers (Basel). 2024;17:66. 39796695 10.3390/cancers17010066 PMC11718901

[132] Sica A, Larghi P, Mancino A, Rubino L, Porta C, Totaro MG, et al. Macrophage polarization in tumour progression. Semin Cancer Biol. 2008;18:349–55. 18467122 10.1016/j.semcancer.2008.03.004

[133] Pittet MJ, Di Pilato M, Garris C, Mempel TR. Dendritic cells as shepherds of T cell immunity in cancer. Immunity. 2023;56:2218–30. 37708889 10.1016/j.immuni.2023.08.014 PMC10591862

[134] Moon CY, Belabed M, Park MD, Mattiuz R, Puleston D, Merad M. Dendritic cell maturation in cancer. Nat Rev Cancer. 2025;25:225–48. 39920276 10.1038/s41568-024-00787-3 PMC11954679

[135] Hoefsmit EP, van Royen PT, Rao D, Stunnenberg JA, Dimitriadis P, Lieftink C, et al. Inhibitor of apoptosis proteins antagonist induces T-cell proliferation after cross-presentation by dendritic cells. Cancer Immunol Res. 2023;11:450–65. 36753604 10.1158/2326-6066.CIR-22-0494

[136] Bhandarkar V, Dinter T, Spranger S. Architects of immunity: how dendritic cells shape CD8 + T cell fate in cancer. Sci Immunol. 2025;10:eadf4726. 39823318 10.1126/sciimmunol.adf4726 PMC11970844

[137] Hao Y, Chung CK, Gu Z, Schomann T, Dong X, Veld RVHI, ’t, et al. Combinatorial therapeutic approaches of photodynamic therapy and immune checkpoint Blockade for colon cancer treatment. Mol Biomed. 2022;3:26. 35974207 10.1186/s43556-022-00086-z PMC9381671

[138] Martinez-Outschoorn UE, Lisanti MP, Sotgia F. Catabolic cancer-associated fibroblasts transfer energy and biomass to anabolic cancer cells, fueling tumor growth. Semin Cancer Biol. 2014;25:47–60. 24486645 10.1016/j.semcancer.2014.01.005

[139] Zhou S, Zhao Z, Wang Z, Xu H, Li Y, Xu K, et al. Cancer-associated fibroblasts in carcinogenesis. J Transl Med. 2025;23:50. 39806363 10.1186/s12967-025-06071-8 PMC11727299

[140] Li Y, Hamad M, Elkord E. Cancer-associated fibroblasts in hepatocellular carcinoma: heterogeneity, mechanisms and therapeutic targets. Hepatol Int. 2025;19:325–36. 39979756 10.1007/s12072-025-10788-5

[141] Mao X, Xu J, Wang W, Liang C, Hua J, Liu J, et al. Crosstalk between cancer-associated fibroblasts and immune cells in the tumor microenvironment: new findings and future perspectives. Mol Cancer. 2021;20:131. 34635121 10.1186/s12943-021-01428-1 PMC8504100

[142] Najafi M, Farhood B, Mortezaee K. Extracellular matrix (ECM) stiffness and degradation as cancer drivers. J Cell Biochem. 2019;120:2782–90. 30321449 10.1002/jcb.27681

[143] Cury SS, Kuasne H, Souza JDS, Muñoz JJM, da Silva JP, Lopes A, et al. Interplay between immune and cancer-associated fibroblasts: a path to target metalloproteinases in penile cancer. Front Oncol. 2022;12:935093. 35928876 10.3389/fonc.2022.935093 PMC9343588

[144] Izar B, Tirosh I, Stover EH, Wakiro I, Cuoco MS, Alter I, et al. A single-cell landscape of high-grade serous ovarian cancer. Nat Med. 2020;26:1271–9. 32572264 10.1038/s41591-020-0926-0 PMC7723336

[145] Charoentong P, Finotello F, Angelova M, Mayer C, Efremova M, Rieder D, et al. Pan-cancer Immunogenomic analyses reveal genotype-immunophenotype relationships and predictors of response to checkpoint Blockade. Cell Rep. 2017;18:248–62. 28052254 10.1016/j.celrep.2016.12.019

[146] Zhu C, Anderson AC, Schubart A, Xiong H, Imitola J, Khoury SJ, et al. The Tim-3 ligand galectin-9 negatively regulates T helper type 1 immunity. Nat Immunol. 2005;6:1245–52. 16286920 10.1038/ni1271

[147] Gieseke F, Kruchen A, Tzaribachev N, Bentzien F, Dominici M, Müller I. Proinflammatory stimuli induce galectin-9 in human mesenchymal stromal cells to suppress T-cell proliferation. Eur J Immunol. 2013;43:2741–9. 23817958 10.1002/eji.201343335

[148] Chowdhury S, Kennedy JJ, Ivey RG, Murillo OD, Hosseini N, Song X, et al. Proteogenomic analysis of chemo-refractory high-grade serous ovarian cancer. Cell. 2023;186:3476–e349835. 37541199 10.1016/j.cell.2023.07.004 PMC10414761

[149] McGranahan N, Rosenthal R, Hiley CT, Rowan AJ, Watkins TBK, Wilson GA, et al. Allele-specific HLA loss and immune escape in lung cancer evolution. Cell. 2017;171:1259–e127111. 29107330 10.1016/j.cell.2017.10.001 PMC5720478

[150] Groom JR, Luster AD. CXCR3 in T cell function. Exp Cell Res. 2011;317:620–31. 21376175 10.1016/j.yexcr.2010.12.017 PMC3065205

[151] Bruand M, Barras D, Mina M, Ghisoni E, Morotti M, Lanitis E, et al. Cell-autonomous inflammation of BRCA1-deficient ovarian cancers drives both tumor-intrinsic immunoreactivity and immune resistance via STING. Cell Rep. 2021;36:109412. 34289354 10.1016/j.celrep.2021.109412 PMC8371260

[152] Dangaj D, Bruand M, Grimm AJ, Ronet C, Barras D, Duttagupta PA, et al. Cooperation between constitutive and inducible chemokines enables T cell engraftment and immune attack in solid tumors. Cancer Cell. 2019;35:885–e90010. 31185212 10.1016/j.ccell.2019.05.004 PMC6961655

[153] Li M, Sun X, Zhao J, Xia L, Li J, Xu M, et al. CCL5 deficiency promotes liver repair by improving inflammation resolution and liver regeneration through M2 macrophage polarization. Cell Mol Immunol. 2020;17:753–64. 31481754 10.1038/s41423-019-0279-0 PMC7331700

[154] Pai C-CS, Huang JT, Lu X, Simons DM, Park C, Chang A, et al. Clonal deletion of tumor-specific T cells by interferon-γ confers therapeutic resistance to combination immune checkpoint Blockade. Immunity. 2019;50:477–e4928. 30737146 10.1016/j.immuni.2019.01.006 PMC6886475

[155] Benci JL, Johnson LR, Choa R, Xu Y, Qiu J, Zhou Z, et al. Opposing functions of interferon coordinate adaptive and innate immune responses to cancer immune checkpoint Blockade. Cell. 2019;178:933–e94814. 31398344 10.1016/j.cell.2019.07.019 PMC6830508

[156] Philips RL, Wang Y, Cheon H, Kanno Y, Gadina M, Sartorelli V, et al. The JAK-STAT pathway at 30: much learned, much more to do. Cell. 2022;185:3857–76. 36240739 10.1016/j.cell.2022.09.023 PMC9815833

[157] Gentric G, Kieffer Y, Mieulet V, Goundiam O, Bonneau C, Nemati F, et al. PML-regulated mitochondrial metabolism enhances chemosensitivity in human ovarian cancers. Cell Metab. 2019;29:156–e17310. 30244973 10.1016/j.cmet.2018.09.002 PMC6331342

[158] Mehibel M, Xu Y, Li CG, Moon EJ, Thakkar KN, Diep AN, et al. Eliminating hypoxic tumor cells improves response to PARP inhibitors in homologous recombination-deficient cancer models. J Clin Invest. 2021;131:e146256. 34060485 10.1172/JCI146256 PMC8266208

[159] Wheeler DA, Takebe N, Hinoue T, Hoadley KA, Cardenas MF, Hamilton AM, et al. Molecular features of cancers exhibiting exceptional responses to treatment. Cancer Cell. 2021;39:38–e537. 33217343 10.1016/j.ccell.2020.10.015 PMC8478080

[160] Zhou Z, Ding Z, Yuan J, Shen S, Jian H, Tan Q, et al. Homologous recombination deficiency (HRD) can predict the therapeutic outcomes of immuno-neoadjuvant therapy in NSCLC patients. J Hematol Oncol. 2022;15:62. 35585646 10.1186/s13045-022-01283-7 PMC9118717

[161] van Wagensveld L, van Baal JOAM, Timmermans M, Gaillard D, Borghuis L, Coffelt SB, et al. Homologous recombination deficiency and Cyclin E1 amplification are correlated with immune cell infiltration and survival in high-grade serous ovarian cancer. Cancers (Basel). 2022;14:5965. 36497449 10.3390/cancers14235965 PMC9738162

[162] Liao G, Jiang Z, Yang Y, Zhang C, Jiang M, Zhu J, et al. Combined homologous recombination repair deficiency and immune activation analysis for predicting intensified responses of anthracycline, cyclophosphamide and taxane chemotherapy in triple-negative breast cancer. BMC Med. 2021;19:190. 34465315 10.1186/s12916-021-02068-4 PMC8408988

[163] Wen Y, Xia Y, Yang X, Li H, Gao Q. CCR8: a promising therapeutic target against tumor-infiltrating regulatory T cells. Trends Immunol. 2025;46:153–65. 39890548 10.1016/j.it.2025.01.001

[164] Zhao Y, Su H, Shen X, Du J, Zhang X, Zhao Y. The immunological function of CD52 and its targeting in organ transplantation. Inflamm Res. 2017;66:571–8. 28283679 10.1007/s00011-017-1032-8

[165] Willingham SB, Volkmer J-P, Gentles AJ, Sahoo D, Dalerba P, Mitra SS, et al. The CD47-signal regulatory protein alpha (SIRPa) interaction is a therapeutic target for human solid tumors. Proc Natl Acad Sci U S A. 2012;109:6662–7. 22451913 10.1073/pnas.1121623109 PMC3340046

[166] Denèfle T, Boullet H, Herbi L, Newton C, Martinez-Torres A-C, Guez A, et al. Thrombospondin-1 mimetic agonist peptides induce selective death in tumor cells: design, synthesis, and structure-activity relationship studies. J Med Chem. 2016;59:8412–21. 27526615 10.1021/acs.jmedchem.6b00781

[167] Kaur S, Roberts DD. Divergent modulation of normal and neoplastic stem cells by thrombospondin-1 and CD47 signaling. Int J Biochem Cell Biol. 2016;81:184–94. 27163531 10.1016/j.biocel.2016.05.005 PMC5097897

[168] Stirling ER, Terabe M, Wilson AS, Kooshki M, Yamaleyeva LM, Alexander-Miller MA, et al. Targeting the CD47/thrombospondin-1 signaling axis regulates immune cell bioenergetics in the tumor microenvironment to potentiate antitumor immune response. J Immunother Cancer. 2022;10:e004712. 36418073 10.1136/jitc-2022-004712 PMC9685258

[169] Maxhimer JB, Soto-Pantoja DR, Ridnour LA, Shih HB, Degraff WG, Tsokos M, et al. Radioprotection in normal tissue and delayed tumor growth by Blockade of CD47 signaling. Sci Transl Med. 2009;1:3ra7. 20161613 10.1126/scitranslmed.3000139 PMC2811586

[170] Moniot A, Schneider C, Chardin L, Yaniz-Galende E, Genestie C, Etiennot M, et al. The CD47/TSP-1 axis: a promising avenue for ovarian cancer treatment and biomarker research. Mol Cancer. 2024;23:166. 39138571 10.1186/s12943-024-02073-0 PMC11323699

[171] Li X, Fang T, Xu S, Jin P, Zhou D, Wang Z, et al. PARP inhibitors promote stromal fibroblast activation by enhancing CCL5 autocrine signaling in ovarian cancer. NPJ Precis Oncol. 2021;5:49. 34108603 10.1038/s41698-021-00189-w PMC8190269

[172] Jin P, Li X, Xia Y, Li H, Li X, Yang Z-Y, et al. Bepotastine sensitizes ovarian cancer to PARP inhibitors through suppressing NF-κB-triggered SASP in cancer-associated fibroblasts. Mol Cancer Ther. 2023;22:447–58. 36780236 10.1158/1535-7163.MCT-22-0396

[173] Pantelidou C, Jadhav H, Kothari A, Liu R, Wulf GM, Guerriero JL, et al. STING agonism enhances anti-tumor immune responses and therapeutic efficacy of PARP Inhibition in BRCA-associated breast cancer. NPJ Breast Cancer. 2022;8:102. 36068244 10.1038/s41523-022-00471-5 PMC9448789

[174] Fenerty KE, Padget M, Wolfson B, Gameiro SR, Su Z, Lee JH, et al. Immunotherapy utilizing the combination of natural killer- and antibody dependent cellular cytotoxicity (ADCC)-mediating agents with Poly (ADP-ribose) Polymerase (PARP) Inhibition. J Immunother Cancer. 2018;6:133. 30486888 10.1186/s40425-018-0445-4 PMC6264611

[175] Sato H, Niimi A, Yasuhara T, Permata TBM, Hagiwara Y, Isono M, et al. DNA double-strand break repair pathway regulates PD-L1 expression in cancer cells. Nat Commun. 2017;8:1751. 29170499 10.1038/s41467-017-01883-9 PMC5701012

[176] Garcia-Diaz A, Shin DS, Moreno BH, Saco J, Escuin-Ordinas H, Rodriguez GA, et al. Interferon receptor signaling pathways regulating PD-L1 and PD-L2 expression. Cell Rep. 2017;19:1189–201. 28494868 10.1016/j.celrep.2017.04.031 PMC6420824

[177] Jiao S, Xia W, Yamaguchi H, Wei Y, Chen M-K, Hsu J-M, et al. PARP inhibitor upregulates PD-L1 expression and enhances cancer-associated immunosuppression. Clin Cancer Res. 2017;23:3711–20. 28167507 10.1158/1078-0432.CCR-16-3215 PMC5511572

[178] Domchek SM, Postel-Vinay S, Im S-A, Park YH, Delord J-P, Italiano A, et al. Olaparib and durvalumab in patients with germline BRCA-mutated metastatic breast cancer (MEDIOLA): an open-label, multicentre, phase 1/2, basket study. Lancet Oncol. 2020;21:1155–64. 32771088 10.1016/S1470-2045(20)30324-7

[179] Drew Y, de Jonge MJ, Hong S, Park Y, Wolfer A, Brown J, et al. Late breaking abstract an open-label, phase II basket study of Olaparib and durvalumab (MEDIOLA): results in germline BRCA-mutated (gBRCAm) platinum-sensitive relapsed (PSR) ovarian cancer (OC). Gynecol Oncol. 2018;149:246–7.

[180] Drew Y, Kaufman B, Banerjee S, Lortholary A, Hong SH, Park YH, et al. Phase II study of olaparib + durvalumab (MEDIOLA): updated results in germline BRCA-mutated platinum-sensitive relapsed (PSR) ovarian cancer (OC). Ann Oncol. 2019;30:v485–6.

[181] Drew Y, Kim J-W, Penson RT, O’Malley DM, Parkinson C, Roxburgh P, et al. Olaparib plus durvalumab, with or without bevacizumab, as treatment in PARP inhibitor-naïve platinum-sensitive relapsed ovarian cancer: a phase II multi-cohort study. Clin Cancer Res. 2024;30:50–62. 37939124 10.1158/1078-0432.CCR-23-2249 PMC10767301

[182] Schram AM, Colombo N, Arrowsmith E, Narayan V, Yonemori K, Scambia G, et al. Avelumab plus Talazoparib in patients with BRCA1/2- or ATM-altered advanced solid tumors: results from JAVELIN BRCA/ATM, an open-label, multicenter, phase 2b, tumor-agnostic trial. JAMA Oncol. 2023;9:29–39. 36394867 10.1001/jamaoncol.2022.5218 PMC9673021

[183] Vinayak S, Tolaney SM, Schwartzberg L, Mita M, McCann G, Tan AR, et al. Open-label clinical trial of niraparib combined with pembrolizumab for treatment of advanced or metastatic triple-negative breast cancer. JAMA Oncol. 2019;5:1132–40. 31194225 10.1001/jamaoncol.2019.1029 PMC6567845

[184] Yap TA, Bardia A, Dvorkin M, Galsky MD, Beck JT, Wise DR, et al. Avelumab plus Talazoparib in patients with advanced solid tumors: the JAVELIN PARP medley nonrandomized controlled trial. JAMA Oncol. 2023;9:40–50. 36394849 10.1001/jamaoncol.2022.5228 PMC9673022

[185] Fanucci KA, Pilat MJ, Shyr D, Shyr Y, Boerner SA, Durecki D, et al. Abstract CT145: Olaparib +/- Atezolizumab in patients with BRCA-mutated (BRCAmt) locally advanced unresectable or metastatic (advanced) breast cancer: an open-label, multicenter, randomized phase II trial. Cancer Res. 2023;83:CT145.

[186] Passiglia F, Righi L, Bironzo P, Listì A, Farinea G, Capelletto E, et al. Niraparib plus dostarlimab in pleural mesothelioma or non-small cell lung cancer harboring HRR mutations: interim results of the UNITO-001 phase II prospective trial. Clin Cancer Res. 2024;30:959–64. 38109438 10.1158/1078-0432.CCR-23-2431

[187] Harano K, Nakao T, Nishio S, Katsuta T, Tasaki K, Takehara K, et al. Neoadjuvant combination treatment of Olaparib and pembrolizumab for patients with HRD-positive advanced ovarian cancer. J Clin Oncol. 2024;42:5545–5545. 10.1158/1078-0432.CCR-25-4887 42531373

[188] Park W, O’Connor C, Chou JF, Schwartz C, Varghese AM, Larsen M, et al. Phase 2 trial of pembrolizumab and Olaparib (POLAR) maintenance for patients (pts) with metastatic pancreatic cancer (mPDAC): two cohorts B non-core homologous recombination deficiency (HRD) and C exceptional response to platinum-therapy. J Clin Oncol. 2023;41:4140–4140.

[189] Kim SI, Joung J-G, Kim Y-N, Park J, Park E, Kim J-W, et al. Durvalumab with or without Tremelimumab plus chemotherapy in HRR non-mutated, platinum-resistant ovarian cancer (KGOG 3045): A phase II umbrella trial. Gynecol Oncol. 2024;182:7–14. 38246047 10.1016/j.ygyno.2023.12.029

[190] Yap TA, Dhawan MS, Hendifar AE, Maio M, Owonikoko TK, Quintela-Fandino M, et al. A phase II study of Olaparib in combination with pembrolizumab in patients with previously treated advanced solid tumors with homologous recombination repair mutation (HRRm) and/or homologous recombination repair deficiency (HRD): KEYLYNK-007. J Clin Oncol. 2020;38:TPS3156–3156.

[191] Jin Y, Du Y, Meng Y, Shao X, Liu X, Mu Y, et al. Results and exploratory biomarker analyses of a phase II study CHANGEABLE: combination of HX008 and niraparib in germ-line-mutated metastatic breast cancer. J Clin Oncol. 2024;42:1084–1084. 10.1002/mco2.70684 PMC13042650 41930344

[192] Stein MN, Dorff TB, Goodman OB, Thomas RA, Silverman MH, Guo M, et al. A phase 2, multicenter, parallel-group, open-label study of Vudalimab (XmAb20717), a PD-1 x CTLA-4 bispecific antibody, alone or in combination with chemotherapy or targeted therapy in patients with molecularly defined subtypes of metastatic castration-resistant prostate cancer. J Clin Oncol. 2022;40:TPS5097–5097.

[193] Schaff L, Fortunato J, Grommes C, Gavrilovic IT, Lin A, Pentsova E, et al. Phase II trial of combination pembrolizumab, olaparib, and Temozolomide for patients with recurrent glioma. J Clin Oncol. 2023;41:TPS2087–2087.

[194] Macarulla T, Castet F, de Mena ML, Garcia-Carbonero R, de Castro EM, Martin AJM, et al. 1686TiP Olaparib and durvalumab (MEDI4736) phase II study in patients with metastatic pancreatic cancer and DNA damage repair genes alterations. Ann Oncol. 2023;34:S923.

[195] Aller EC, Hanna G, Villar MV, Even C, Moreno V, Kim C et al. 589 Xevinapant plus nivolumab in patients with advanced solid tumors who progressed on prior anti–PD-1/PD-L1 treatment: results of a dose-optimization, exploratory phase 1b/2 trial. J Immunother Cancer. 2022 [cited 2025 Feb 14];10. Available from: https://jitc.bmj.com/content/10/Suppl_2/A616

[196] Harter P, Bidziński M, Colombo N, Floquet A, Rubio Pérez MJ, Kim J-W, et al. DUO-O: a randomized phase III trial of durvalumab (durva) in combination with chemotherapy and Bevacizumab (bev), followed by maintenance durva, Bev and Olaparib (olap), in newly diagnosed advanced ovarian cancer patients. J Clin Oncol. 2019;37:TPS5598–5598.

[197] Fujiwara K, Chou H-H, Kim J-W, Tan DS, Tamura K, Katsumata N, et al. 263TiP - ATHENA (GOG-3020/ENGOT-ov45): a randomised, double-blind, placebo-controlled phase III study of the Poly (ADP-ribose) Polymerase (PARP) inhibitor rucaparib + the PD-1 inhibitor nivolumab following frontline platinum-based chemotherapy in ovarian cancer. Ann Oncol. 2019;30:ix88–9.

[198] Domchek S, Bang Y-J, Coukos G, Kobayashi K, Baker N, McMurtry E, et al. MEDIOLA: a phase I/II, open-label trial of Olaparib in combination with durvalumab (MEDI4736) in patients (pts) with advanced solid tumours. Ann Oncol. 2016;27:vi377.

[199] van Wilpe S, Kloots ISH, Slootbeek PHJ, den Brok M, Westdorp H, Franken MD, et al. Ipilimumab with nivolumab in molecularly selected patients with castration-resistant prostate cancer: primary analysis of the phase II INSPIRE trial☆. Ann Oncol. 2024;35:1126–37. 39293514 10.1016/j.annonc.2024.09.004

[200] Hyman DM, Zelnak AB, Bauer TM, Ulahannan SV, Ford JM, Cesari R, et al. JAVELIN BRCA/ATM: a phase 2 trial of avelumab (anti–PD-L1) plus Talazoparib (PARP inhibitor) in patients with advanced solid tumors with a BRCA1/2 or ATM defect. J Clin Oncol. 2019;37:TPS2660–2660.

[201] Konstantinopoulos PA, Waggoner SE, Vidal GA, Mita MM, Fleming GF, Holloway RW, et al. TOPACIO/Keynote-162 (NCT02657889): a phase 1/2 study of niraparib + pembrolizumab in patients (pts) with advanced triple-negative breast cancer or recurrent ovarian cancer (ROC)—Results from ROC cohort. J Clin Oncol. 2018;36:106–106.

[202] Konstantinopoulos PA, Waggoner S, Vidal GA, Mita M, Moroney JW, Holloway R, et al. Single-arm phases 1 and 2 trial of niraparib in combination with pembrolizumab in patients with recurrent platinum-resistant ovarian carcinoma. JAMA Oncol. 2019;5:1141–9. 31194228 10.1001/jamaoncol.2019.1048 PMC6567832

